# Growth–Mortality Coordination Differs Among Xerophytic Versus Mesophytic Tree Species During Severe Drought

**DOI:** 10.1111/gcb.70260

**Published:** 2025-06-02

**Authors:** Michael C. Benson, Taehee Hwang, Justin T. Maxwell, Richard P. Phillips, Kimberly A. Novick

**Affiliations:** ^1^ O'Neill School of Public and Environmental Affairs Indiana University Bloomington Bloomington Indiana USA; ^2^ Department of Plant Biology University of Illinois at Urbana‐Champaign Urbana Illinois USA; ^3^ Department of Geography Indiana University Bloomington Bloomington Indiana USA; ^4^ Department of Biology Indiana University Bloomington Bloomington Indiana USA

**Keywords:** climate change, drought, forest inventory and analysis, growth, management, mortality

## Abstract

Forest composition is changing, yet the consequences for terrestrial carbon cycling are unclear. In the eastern United States, water‐demanding “mesophytic” tree species are replacing “xerophytic” oaks (*Quercus* spp.) and hickories (*Carya* spp.), raising concerns that forest productivity will become increasingly sensitive to more frequent and severe drought conditions predicted for the region. However, we have a limited understanding of the extent to which the mortality risk of xerophytes versus mesophytes is coordinated with their growth sensitivity during drought. Here, we evaluated growth and mortality dynamics for 20 abundant eastern United States tree species following a severe drought in the summer of 2012. We synthesized data from ~4500 forest inventory plots and used an approach that quantified relative drought responses between co‐located trees to minimize impacts from environmental heterogeneity. We found that mesophytes were just as likely to perish as co‐occurring xerophytes but were more sensitive to drought in terms of diminished growth. These findings suggest that xerophytic decline is likely to lead to reduced carbon uptake during drought and that management efforts to conserve oak‐hickory stands will be decisive to sustain the carbon mitigation potential of these forests. However, we also found that growth‐mortality relationships differed between functional groups. Among xerophytes, growth and survival during drought were decoupled. Among mesophytes, there was a high degree of coordination, where species that experienced greater mortality also experienced greater growth reductions. Therefore, mesophytes with high growth sensitivity to water deficits are likely to be the most vulnerable to drought‐driven die‐off events moving forward.

## Graphical abstract

In the eastern United States, water‐demanding “mesophytic” trees are replacing “xerophytic” oaks and hickories. In this article, we evaluated the terrestrial carbon consequences of this ongoing compositional shift by quantifying relative drought‐driven growth and mortality responses between co‐located trees. We found that growth and mortality sensitivities were strongly coordinated among mesophytes but not xerophytes. Additionally, mesophytes and xerophytes experienced similar degrees of mortality but mesophytes were more sensitive in terms of growth. These findings suggest that declining xerophyte abundance will likely have a larger effect on diminished carbon uptake during future droughts as opposed to increased carbon losses via mortality.

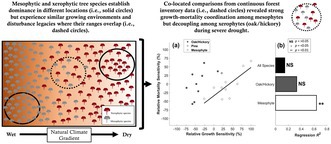

## 1 Introduction

The trade‐off between tree growth and mortality is a central tenet of plant life history theory (Grubb 1977; Wright et al. 2010). The trade‐off emerges because tree species possess diverse functional traits that enable a continuum of resource acquisition strategies (Reich 2014). On one end, trees with traits that facilitate rapid growth readily establish dominance when resources are abundant but are highly sensitive to stress or quickly die when resources become scarce. On the other end, trees with slower growth strategies invest in traits that limit the adverse consequences of resource decline but are vulnerable to being outcompeted by faster‐growing neighbors when resources are non‐limiting (Wright et al. 2010; Adler et al. 2014). Where plants fall along this spectrum is a strong determinant of terrestrial carbon balance and is directly related to the composition of species and traits within sympatric forest communities (Anderegg et al. 2016; Spînu et al. 2020; Trugman et al. 2020; Alexander et al. 2021). To assess how forest productivity will respond to increased hydroclimate variability in the future, it is important to understand how growth and mortality relationships evolve with forest composition change.

One generalizable way to characterize forest composition change is by focusing on the relative abundance of mesophytic versus xerophytic species. Mesophytes are shade‐tolerant species which possess traits that help maximize carbon uptake and establishment in water‐rich environments but may promote higher sensitivity to water deficits; in contrast, xerophytes are shade‐intolerant species which possess traits that maintain carbon uptake and survival in water‐limited environments but are less able to capitalize on periods of abundant water supply (Valladares and Niinemets 2008; Lombardini and Rossi 2019; Alexander et al. 2021). In some places, such as the western United States, climate change has increased the occurrence of drought events in ways that are favoring the establishment and survival of xerophytic species (Allen and Breshears 1998; Trugman et al. 2020). In other places, like the eastern United States, forest management has driven ongoing increases in the relative abundance of mesophytes (Fei et al. 2011; Alexander et al. 2021). The consequences of these compositional shifts for forest productivity depend strongly on the distinct characteristics of these two groups, and the extent that their drought‐tolerant niches will persist in a future defined by more frequent and severe drought events (Augusto et al. 2025).

It is logical to assume that mesophytes are particularly vulnerable to extreme drought stress. Mesophytes establish dominance in water‐rich environments by overtopping their neighbors, owing to their higher relative allocation to aboveground biomass (Wright et al. 2010; Alexander et al. 2021). This higher investment in photosynthetic tissue enables mesophytes to grow faster than xerophytes, but their subsequent reliance on shallow rooting profiles makes their productivity sensitive to transient water deficits (Meinzer et al. 2013). Diminished productivity often precedes forest mortality (DeSoto et al. 2020) and prolonged declines in carbon uptake can exhaust mesophyte's ability to accommodate their high canopy resource demands (Buckley et al. 2017; Jump et al. 2017). However, it is possible that the same traits which limit their fitness in xeric landscapes may make them less likely to die during hotter and drier droughts. Recent work has highlighted that mesophyte's tendency to down‐regulate their carbon uptake during drought may allow them to maintain a greater window of safety from catastrophic xylem embolism and desiccation (Gu et al. 2015; Kannenberg, Novick, et al. 2019; Benson et al. 2022; Novick et al. 2022). Thus, how the unique trait assemblages of mesophytes versus xerophytes integrate to govern the relationship between growth and mortality during severe drought events remains an open question.

Though forest composition change is occurring globally (Feeley et al. 2011; Bhatta and Vetaas 2016; Spînu et al. 2020; Trugman et al. 2020; Alexander et al. 2021) the eastern United States represents an important case study to evaluate how drought‐driven growth and mortality dynamics differ among xerophytes and mesophytes. Across the region, xerophytic oak‐hickory (*Quercus‐Carya* spp.) type forests have disproportionately contributed to key ecosystem services related to carbon uptake and storage (Heath et al. 2002; Cavender‐Bares 2016). Unfortunately, these services are threatened by the ongoing decline of regional oak‐hickory abundance (Pierce et al. 2006; Fei et al. 2011; Novick et al. 2022). Throughout the 20th century, changing conditions have favored the establishment of mesophytic species such as maples (*Acer* spp.), American beech ( *Fagus grandifolia* ) and tulip poplar ( *Liriodendron tulipifera* ) (Alexander et al. 2021). The loss of oak‐hickory forests has been attributed to multiple drivers, including fire suppression and wet climate conditions during the last century that have inhibited oak‐hickory regeneration (McEwan et al. 2011; Pederson et al. 2015). Regardless of the cause, the increasing abundance of mesophytes has raised concerns that the productivity of eastern United States forests, a globally important terrestrial carbon sink (Xiao et al. 2011; FAO and UNEP 2020), may be especially vulnerable to future severe droughts caused by rising temperatures and altered precipitation regimes (Brzostek et al. 2014; Coble et al. 2017; Iverson et al. 2018; Au et al. 2020; Novick et al. 2022).

Oak‐hickory forests have long been viewed as drought resistant, owing to their abundance in xeric landscapes (Abrams 2003; Arthur et al. 2015) and propensity to sustain carbon uptake and growth during hydrologic stress (Niinemets and Valladares 2006; Gu et al. 2015; Roman et al. 2015; Hu et al. 2017; Denham et al. 2021). However, episodic drought‐mortality in oak‐hickory forests has been increasingly reported in recent decades (Fan et al. 2012; Haavik et al. 2015; Radcliffe et al. 2021), which complicates our understanding of the carbon consequences of this ongoing compositional shift. If xerophytic oaks and hickories are indeed more prone to drought‐driven mortality, their beneficial impacts on forest carbon cycling may be diminished (Xu et al. 2012), as die‐off events not only reduce forest productivity (Liu et al. 2023) but emit terrestrial carbon back into the atmosphere (van der Molen et al. 2011).

In this study, we quantify the drought‐driven growth and mortality sensitivities for a wide range of eastern United States forest tree species. We leverage the wealth of data accumulating in the USDA Forest Inventory and Analysis (FIA) survey, which provides spatially explicit and co‐located observations of stem loss and growth across thousands of permanent plots (Gray et al. 2012). While intense droughts are expected to become more frequent across eastern United States forests as the climate continues to warm (Zhao et al. 2020), drought events have been relatively rare in the region since the standardization of FIA sampling schemes (Maxwell and Harley 2017). However, the central hardwood ecoregion of the eastern United States experienced an exceptional drought in the summer of 2012 (Mallya et al. 2013), providing a unique opportunity to assess growth and mortality dynamics of xerophytes versus mesophytes during one of the most extreme drought disturbances to affect the region during the last century.

Our analyses are guided by the following questions:

1. Are xerophytic oak and hickory species more (or less) vulnerable to severe drought in terms of growth and survival than mesophytes?
2. To what extent do xerophytes and mesophytes differ in their relationship between growth and mortality during severe drought?

Collectively, answers to these questions will generate timely and important information regarding forest management in the face of climate change (Fralish 2004; Holzmueller et al. 2014). Though previous studies have addressed similar questions in eastern United States forests (e.g., Niinemets and Valladares 2006; D'Orangeville et al. 2018; Au et al. 2020; Maxwell et al. 2024), a widespread focus on growth, rather than mortality, has neglected to consider the extent to which short‐term gains via growth may be offset by mortality and the loss of standing carbon stock. By evaluating the relationship between growth and survival for xerophytic oak/hickory species and mesophytes during severe drought, we gain a more holistic understanding of the potential carbon costs of ongoing forest compositional shifts.

## 2 Methods

### 2.1 Data, Study Area, and Species

Data on tree growth and mortality were accessed from the USDA Forest Service Forest Inventory and Analysis (FIA) Program. The FIA survey is a long‐term record on the status of United States forests based on repeated field sampling of plot‐level silvicultural metrics including tree species, their diameter at breast height (DBH), and other site characteristics (Bechtold and Patterson 2005). FIA plots consist of a cluster of four ~42 m^2^ subplots located 36.6 m away from a central plot at 0‐, 120‐, and 240° azimuth (Gray et al. 2012). Plot locations are distributed across the continental United States with approximately one sample location every 2428 ha, but often at higher density in contiguously forested public lands. FIA records extend back to the 1960s, but prior to 2000 individual states sampled plots periodically and at asynchronous intervals (Fei et al. 2011; Gray et al. 2012).

**FIGURE 1:**
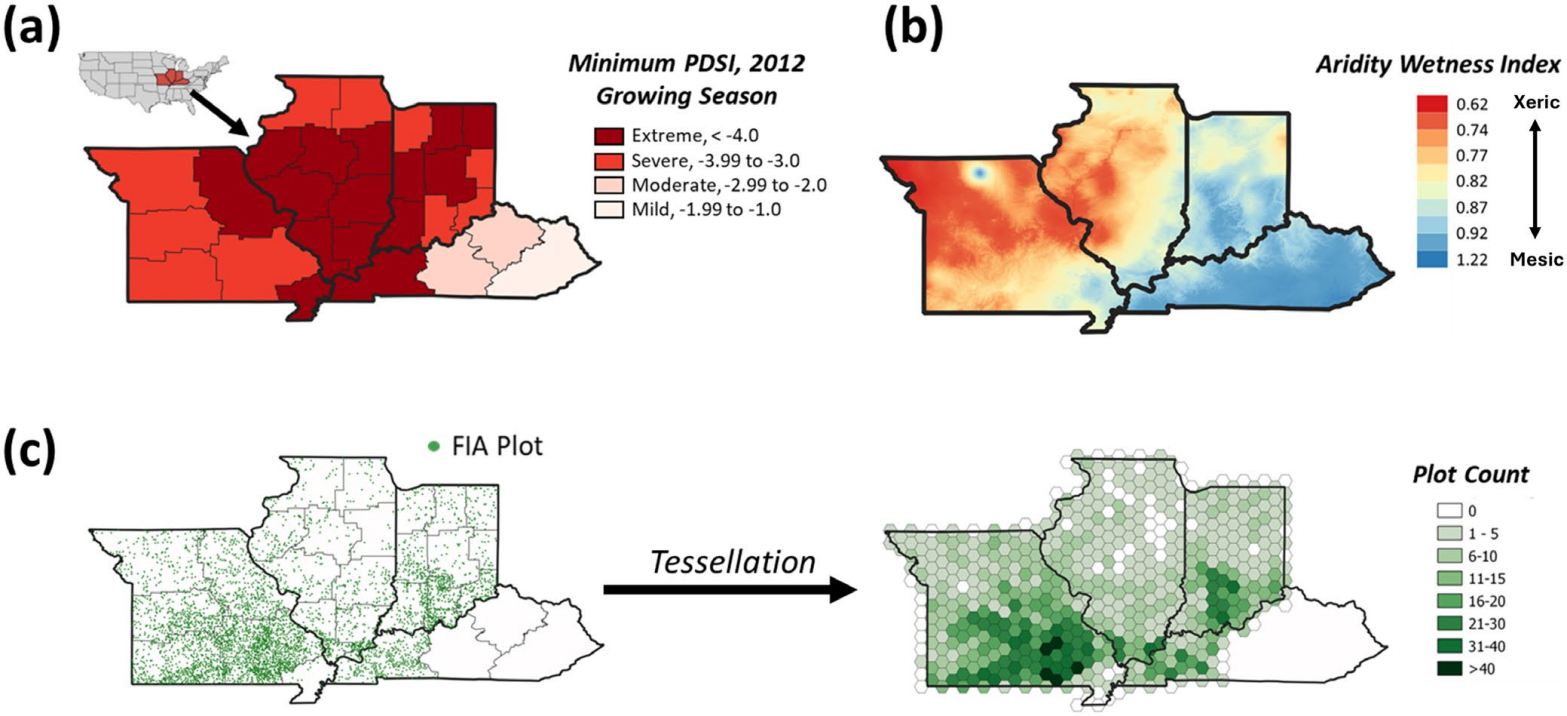
Drought exposure, historical climate conditions, and Forest Inventory and Analysis plot distribution across the study area. Panel (a) is minimum monthly Palmer Drought Severity Index (PDSI) values across climate zones during the 2012 growing season (March—October). Panel (b) is long‐term aridity‐wetness index at 30 arc‐second spatial resolution from 1970 to 2000 (Trabucco and Zomer 2018). The aridity‐wetness index denotes the ratio of mean annual precipitation (informed by Worldclim2 interpolated weather station data) to mean annual evapotranspiration (derived by the FAO Penman‐Monteith method); for more detail on the calculation of the aridity‐wetness index, see: Trabucco and Zomer 2018). Panel (c) is the plot locations and the tessellation approach whereby individual plots were reclassified and aggregated to uniform ~875 km^2^ hexagons. Map lines delineate study areas and do not necessarily depict accepted national boundaries.

In this study, we quantified tree growth and mortality from 2000 to 2018 across ~4500 FIA plots that experienced “Severe” or “Extreme” water stress (Svoboda et al. 2002) during the exceptional 2012 drought (Mallya et al. 2013) (Figure 1a). Historically, damaging droughts in the central hardwoods have been relatively infrequent. Over the past century, severe droughts prior to 2012 all occurred before 1970, the majority of which were in the “Dust Bowl” era of the 1930s (Brandt et al. 2014). Additionally, the 2012 drought event was especially damaging because precipitation was reduced relatively early in the growing season. In southern Indiana, for example, only 23 mm of precipitation occurred in the months of June and July, which was less than 10% of the historical average (Roman et al. 2015). In other years, pronounced soil moisture deficits typically do not develop until late summer or autumn when trees have largely stopped growing (Yi et al. 2017).

The study region is broadly characterized by temperate climates, but with a pronounced east–west gradient (Figure 1b). We focused on the most abundant tree species in the region that exceeded 10,000 observations in the FIA subset and had a DBH greater than 12.7 cm (Fei et al. 2011; Jo et al. 2019). We excluded timber‐harvested plots and sub‐canopy tree species such as flowering dogwood ( *Cornus florida* ) and eastern red cedar ( *Juniperus virginiana* ). Ash species ( *Fraxinus pennsylvanica* and *Fraxinus americana* ) were also removed due to widespread mortality associated with the spread of the emerald ash borer (Pugh et al. 2011). In total, our analyses included 20 highly abundant canopy‐dominant tree species, representing ~80% of regional FIA observations during the study period (Table 1).

**TABLE 1:** Study species, functional groups, and number of observations in the Forest Inventory and Analysis subset from 2000 to 2018 used in this study.

Species name	Species code	Common name	Functional group	FIA inventory observations
*Quercus alba*	QUAL	White Oak	Oak	62,224
*Acer saccharum*	ACSA	Sugar Maple	Mesophyte	37,790
*Quercus velutina*	QUVE	Black Oak	Oak	37,030
*Quercus stellata*	QUST	Post Oak	Oak	31,186
*Acer rubrum*	ACRU	Red Maple	Mesophyte	25,730
*Liriodendron tulipifera*	LITU	Tulip Poplar	Mesophyte	21,600
*Ulmus americana*	ULAM	American Elm	Mesophyte	21,126
*Carya ovata*	CAOV	Shagbark Hickory	Hickory	15,829
*Pinus echinata*	PIEC	Shortleaf Pine	Pine	15,416
*Carya glabra*	CAGL	Pignut Hickory	Hickory	14,563
*Prunus serotina*	PRSE	Black Cherry	Mesophyte	14,213
*Quercus rubra*	QURU	Red Oak	Oak	13,821
*Sassafras albidum*	SAAL	Sassafras	Mesophyte	13,667
*Celtis occidentalis*	CEOC	Hackberry	Mesophyte	13,483
*Juglans nigra*	JUNI	Black Walnut	Mesophyte	13,406
*Carya alba*	CAAL	Mockernut Hickory	Hickory	13,124
*Nyssa sylvatica*	NYSY	Black Gum	Mesophyte	11,263
*Carya texana*	CATE	Black Hickory	Hickory	10,968
*Quercus coccinea*	QUCO	Scarlet Oak	Oak	10,530
*Fagus grandifolia*	FAGR	American Beech	Mesophyte	10,286

Although our region of interest included an appreciable number of FIA plots, the mean number of canopy dominant trees at the plot level (independent of species) was 18.54 (±10.71 std), and it was common to have just 1 or 2 individuals of any given species present in a single plot. Because small stem counts can skew estimates of growth and especially mortality (Sheil et al. 1995; Zhou et al. 2021) we applied a tessellation scheme (Fei et al. 2017; Jo et al. 2019), whereby FIA plots were regionally aggregated to 560 uniformly spaced hexagons (with an area equal to ~875 km^2^) (Figure 1c). The coordinates of each hexagon center and their species composition are reported in the (Table S1). Additionally, we used a spatial smoothing measure by interpolating plot information from surrounding regions; the unit of analysis for this study was defined as the individuals in each hexagon plus the sum of those in their surrounding hexagons (Figure S1).

### 2.2 Analytical Approach

Relying on FIA data to understand how growth and mortality vary across species and functional groups requires a careful approach. Forest inventory databases have been essential to understanding changing forest demographics (Fei et al. 2011; Pugh et al. 2011; Trugman et al. 2020) and estimating the quantity of stem loss following disturbance (Kromroy et al. 2008; Klos et al. 2009; Thompson 2009; Venturas et al. 2021). However, it is important to recognize that drought impacts on the landscape are highly sensitive to localized environmental factors, such as aridity (Lévesque et al. 2014; Jump et al. 2017), soil texture (Redmond et al. 2015), and disturbance legacies (Kannenberg et al. 2020; Knapp et al. 2021). The xerophytic and mesophytic nature of our study species means that their range and degree of coexistence often differ (Figures S2 and S3) as historical climate conditions have shaped their establishment (Figure S4). Because the densities of these co‐dominant trees are not spatially uniform, species can differ in their degree of drought exposure or be differentially sensitive to physiological stress during drought (Figure S5). Collectively, these environmental factors complicate our ability to compare growth and mortality responses from inventory data.

**FIGURE 2:**
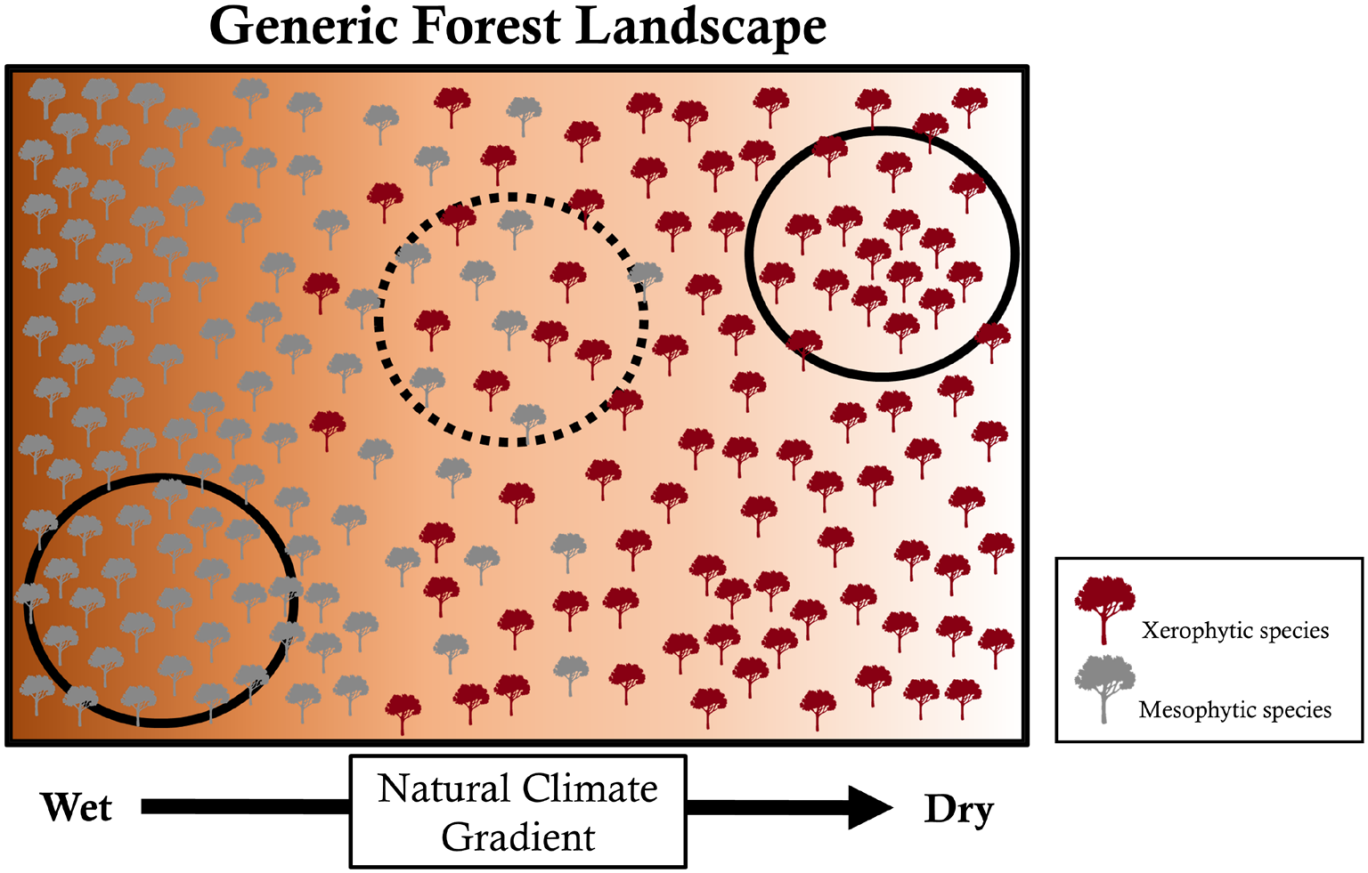
Conceptual figure demonstrating the co‐located filtering approach used to minimize confounding biases associated with range distributions and environmental factors. Across a natural climate gradient, xerophytic and mesophytic species are most abundant in different locations. Rather than comparing drought responses in regions where these species were spatially separated and subjected to different pedo‐environmental factors (solid black circles), species were *only* compared in overlapping regions (dashed black circle) where they experienced similar disturbance legacies and growing environments.

To overcome these challenges, we developed a novel methodology that applied traditional geographic axioms (Tobler's First Law of Geography; Tobler 1969) to minimize confounding biases associated with range distributions and environmental factors. This approach allowed us to quantify relative drought impacts *only* in regions where specific species co‐occurred (Figure 2). In this manner, the complications of site‐to‐site variation driven by topo‐climatic variability are reduced because drought responses are only compared between individuals that have been subjected to similar degrees of drought exposure, disturbance legacies, and growing environments (Au and Maxwell 2022; Novick et al. 2022).

### 2.3 Growth Sensitivity Analyses

To normalize the impact of site‐to‐site environmental variation, we evaluated relative growth responses associated with the 2012 drought in hexagons where species pairs overlapped, adopting an approach that relies on relative comparisons among co‐located species (Figure 2). We first quantified species‐specific growth at the hexagon‐level as a relative growth rate for species i (${\mathrm{RGR}}_{i}$) (% growth/year) among individuals (Brzostek et al. 2014), expressed as:

(1) $${\mathrm{RGR}}_{i}=\left[\frac{\left({\mathrm{BA}}_{i,1}-{\mathrm{BA}}_{i,0}\right)}{{\mathrm{BA}}_{i,0}}\right]\times 100$$

where ${\mathrm{BA}}_{i,0}$ is the basal area (the cross‐sectional area of tree stems (m^2^)) at the initial inventory and ${\mathrm{BA}}_{i,1}$ is the basal area at the next inventory. Because the species in our study region exhibit different intrinsic growth rates (Latham 1992), the impact of drought on ${\mathrm{RGR}}_{i}$ was characterized as a rate change parameter, $∆{\mathrm{RGR}}_{i}$ (% growth/year), whereby ${\mathrm{RGR}}_{i}$ after 2012 was subtracted by the ${\mathrm{RGR}}_{i}$ of the conspecific individuals occupying the same hexagon prior to drought disturbance. Specifically, $∆{\mathrm{RGR}}_{i}$ was quantified as:

(2) $$∆{\mathrm{RGR}}_{i}={\mathrm{RGR}}_{i,\text{post}\_\text{drought}}-{\mathrm{RGR}}_{i,\mathrm{pre}\_\text{drought}}$$

where ${\mathrm{RGR}}_{i,\text{post}\_\text{drought}}$ is species‐specific relative tree growth following drought (when ${\mathrm{BA}}_{i,1}$ was sampled during the years 2012–2018; Equation 1) and ${\mathrm{RGR}}_{i,\mathrm{pre}\_\text{drought}}$ is the relative tree growth of the same species and locations from two successive inventories during a non‐droughted period (i.e., when ${\mathrm{BA}}_{i,1}$ was sampled during the years 2000–2011; Equation 1).

Next, we used an iterative pairwise difference approach to compare corrected growth rates between species. The relative growth parameter, $g_{r\_\mathrm{ij}}$ (% growth/year), was expressed as:

(3) $$g_{r\_\mathrm{ij}}=∆{\mathrm{RGR}}_{i}-∆{\mathrm{RGR}}_{j}$$

where $∆{\mathrm{RGR}}_{i}$ is ∆RGR of a specific species (i.e., species i) and $∆{\mathrm{RGR}}_{j}$ is ∆RGR of one of the other 19 study species (Table 1) that occupied the same hexagon. We determined whether drought‐driven $g_{r\_\mathrm{ij}}$ was different between species pairs using a one sample *t‐*test (*α* = 0.05). A negative $g_{r\_\mathrm{ij}}$ indicates species i's growth was more strongly reduced by the 2012 drought than species j while a positive $g_{r\_\mathrm{ij}}$ indicates that species i experienced lower relative growth reductions; a $g_{r\_\mathrm{ij}}$ equal to or near zero indicates the drought impact on growth between species pairs i and j was equivalent.

To compare which tree species' growth was more (or less) sensitive to drought after correcting for the influence of local environmental factors, we synthesized $g_{r\_\mathrm{ij}}$ across all species pairs by quantifying a relative sensitivity metric. This metric was characterized as a species‐specific percentile difference of the number of species which experienced greater or more reduced relative growth than their neighbors. Specifically, relative growth sensitivity (%) was quantified as:

(4) $$\text{Relative}\;{\text{growth sensitivity}}_{i}=\left[\frac{\left(n_{g_{r\_\mathrm{ij}}<0}\right)-\left(n_{g_{r\_\mathrm{ij}}>0}\right)}{n_{\mathrm{tot}}}\right]\times 100$$

Where $n_{g_{r\_\mathrm{ij}}<0}$ are the number of co‐occurring species that experienced lower growth reductions and $n_{g_{r}\_\mathrm{ij}>0}$ are the number of species whose growth was more limited by drought. $n_{\mathrm{tot}}$ is the number of species that had overlapping ranges. A positive relative growth sensitivity value indicates growth was more strongly reduced by drought while a negative value indicates greater growth tolerance. We then compared the mean differences of relative growth sensitivity between the xerophytic oak/hickory and mesophyte groups (Table 1) using a two‐sample *t*‐test at the *α* = 0.05 significance level.

### 2.4 Mortality Sensitivity Analyses

Our approach to estimate species‐level mortality responses was similar to the approach used to characterize growth sensitivity (Section 2.3). Species‐specific mortality was quantified as a stem loss rate ($m_{i}$) (% stem loss/year) using the equation provided by Sheil et al. (1995):

(5) mi=1−Ni,1Ni,01t×100

where $N_{i,0}$ is the number of live stems (the standing tree species denoted as living in the FIA survey) at the initial inventory (at year = $t_{0}$) and $N_{i,1}$ is the number of live stems at the next inventory (at year = $t_{1})$. The variable t (years) is the difference in time between inventory periods, and equal to $t_{1}-t_{0}$.

It is important to recognize that assessing drought‐driven mortality as a change in live stem counts between inventories (i.e., Equation 5) can be sensitive to recruitment and drought‐independent mortality agents (i.e., vulnerability to windthrow, pests, disease, etc.). To account for this, we evaluated the impact of drought as a change in stem loss rate ($∆m_{i}$) (% stem loss/year), where species‐specific $m_{i}$ after 2012 were subtracted by $m_{i}$ quantified in the same hexagon during previous non‐drought periods. The $∆m_{i}$ equation took the following form:

(6) $$∆m_{i}=m_{i,\text{post}\_\text{drought}}-m_{i,\mathrm{pre}\_\text{drought}}$$

where $m_{i,\text{post}\_\text{drought}}$ is a species‐specific drought‐driven stem loss rates (when $N_{i,1}$ was sampled during the years 2012–2018; Equation 5) and $m_{i,\mathrm{pre}\_\text{drought}}$ is the stem loss rate for the same species and locations from two successive inventories during a non‐droughted period (when $N_{i,1}$ was sampled during the years 2000–2011; Equation 5).

Next, we applied an analogous iterative pairwise difference approach (i.e., Equation 3) to compare corrected mortality responses among individuals that co‐occurred in the same hexagons. The relative mortality, $m_{r\_\mathrm{ij}}$ (% stem loss/year), equation took the following form:

(7) $$m_{r\_\mathrm{ij}}=∆m_{i}-∆m_{j}$$

Where $∆m_{i}$ is ∆m of a specific species (i.e., species i) and $∆m_{j}$ is ∆m of one of the other 19 study species (Table 1) that occupied the same hexagon. We determined whether drought‐driven $m_{r\_\mathrm{ij}}$ was different between species pairs using a one sample *t‐*test (*α* = 0.05). A positive $m_{r\_\mathrm{ij}}$ indicates stem loss was greater for species i than species j, a negative $m_{r\_\mathrm{ij}}$ indicates that stem loss was lower for species i, and a $m_{r\_\mathrm{ij}}$ equal to or near zero indicates the impact of drought on stem loss between species pairs i and j was equivalent.

We then synthesized $m_{r\_\mathrm{ij}}$ across all species pairs to characterize an analogous relative drought‐tolerant metric for mortality. Relative mortality sensitivity (%) was thus quantified as:

(8) $$\text{Relative}\;{\text{mortality sensitivity}}_{i}=\left[\frac{\left(n_{m_{r\_\mathrm{ij}}>0}\right)-\left(n_{m_{r\_\mathrm{ij}}<0}\right)}{n_{\mathrm{tot}}}\right]\times 100$$

where $n_{m_{r\_\mathrm{ij}}>0}$ are the number of co‐occurring species that experienced lower drought‐driven stem loss change and $n_{m_{r\_\mathrm{ij}}<0}$ are the number of species that experienced greater drought‐driven stem loss change. $n_{\mathrm{tot}}$ is the number of species that had overlapping ranges. A positive relative mortality sensitivity value indicates a species experienced greater mortality than their neighbors while a negative one indicates greater survival. Likewise, the absolute magnitude reflects the strength of stem loss response. Mean differences between the xerophytic oak/hickory and mesophyte groups (Table 1) were compared using a two‐sample *t*‐test at the *α* = 0.05 significance level. Additionally, we used regression analyses to assess the relationship between relative growth and mortality sensitivities (Equations 4 and 8, respectively). All analyses and statistical tests were conducted in MATLAB software v. R2019a (MathWorks Inc.; Natick, MA, USA).

## 3 Results

**FIGURE 3:**
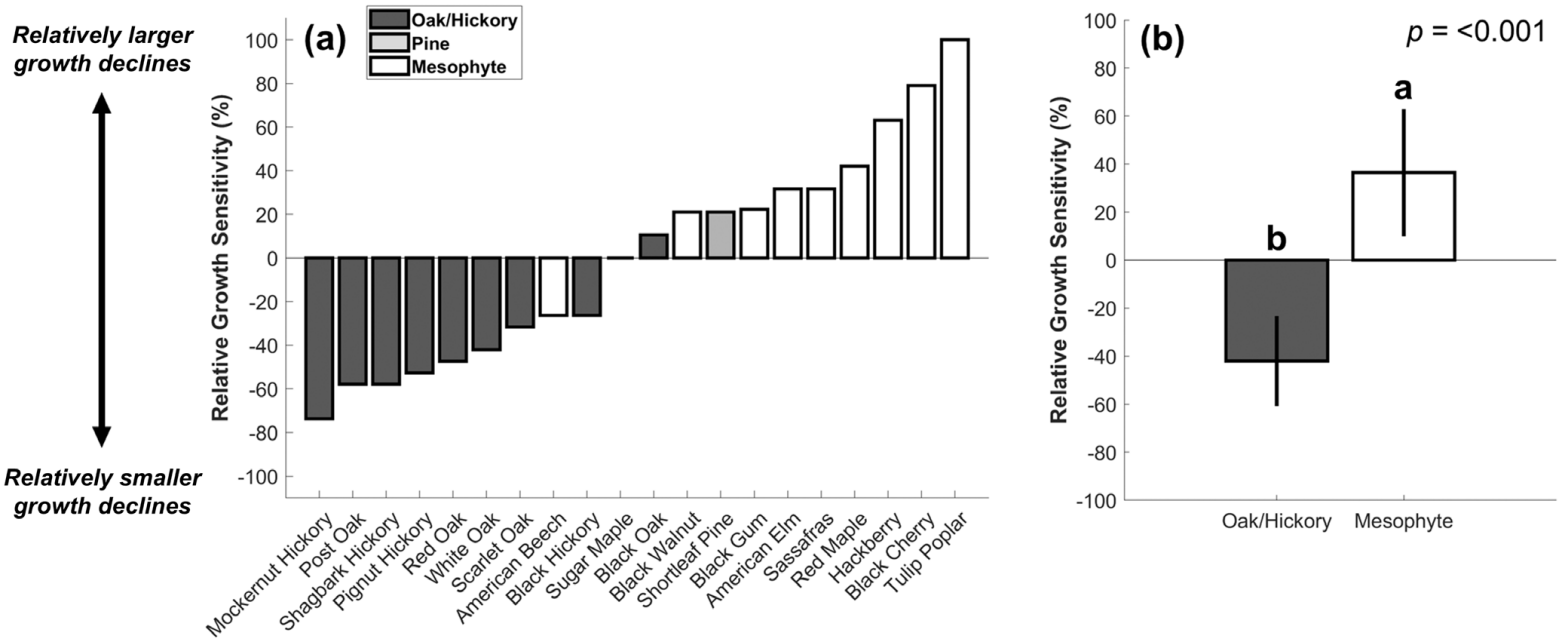
Relative growth sensitivity (Equation 4) across species (panel (a)) and functional groups (panel (b)). Error bars denote 95% confidence intervals and letters above bars denote significant mean differences from a two‐tailed *t*‐test (*α* = 0.05).

Relative growth sensitivities to the 2012 drought among co‐located individuals revealed strong functional differences between xerophytic oak/hickory versus mesophytic species (Figure 3). Except for black oak, all oak/hickory species had a negative relative growth sensitivity metric value (Figure 3a), indicating that their productivity was inhibited by drought to a lesser extent than their neighbors. Likewise, the majority of mesophytes had a positive relative growth sensitivity metric value (Figure 3a). Sugar maple and American beech had similar responses to many co‐occurring trees and were among the most tolerant mesophytes in terms of growth. However, other mesophytic species like tulip poplar, hackberry, and black cherry, were notably sensitive and experienced the greatest productivity declines (Figure 3a). The iterative growth comparisons between individual species pairs that informed the relative growth sensitivity metric ($g_{r\_\mathrm{ij}}$; Equation 3) are reported in the (Figure S6).

**FIGURE 4:**
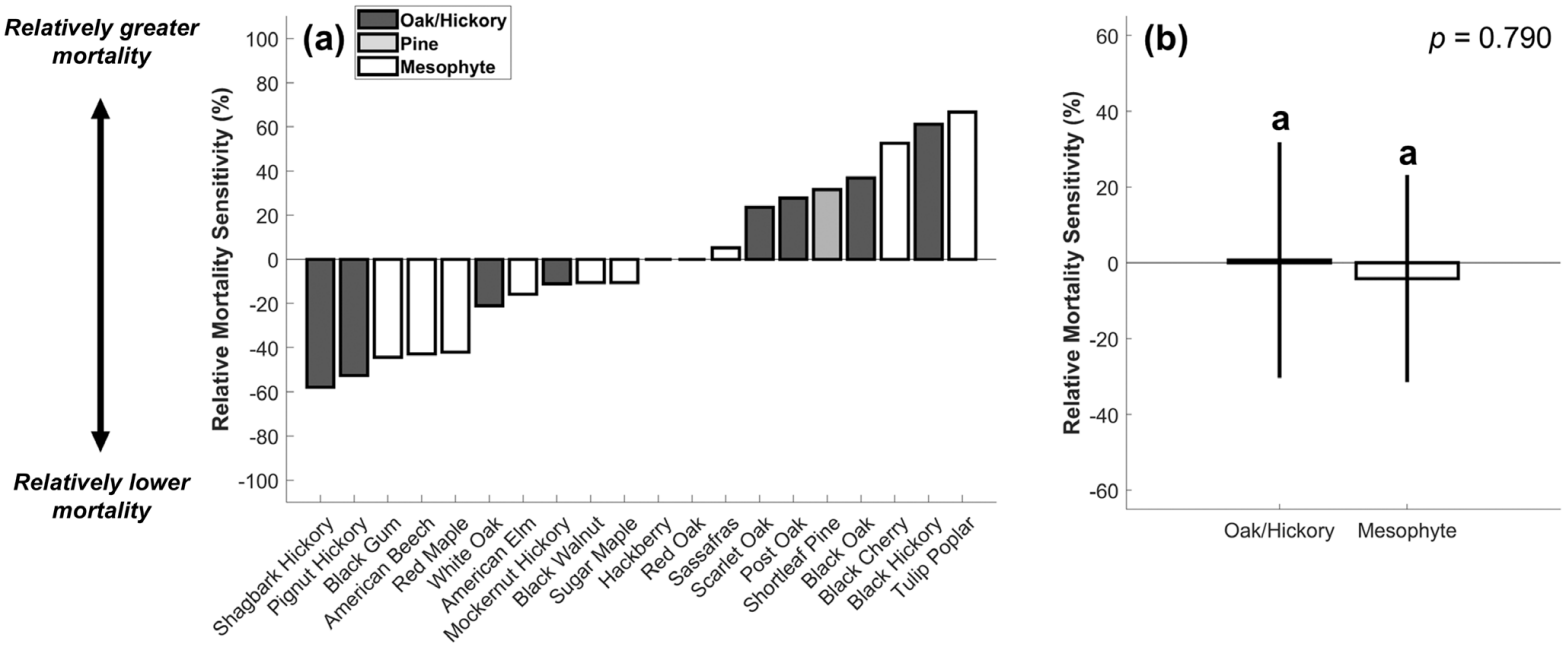
Relative mortality sensitivity (Equation 8) across species (panel (a)) and functional groups (panel (b)). Error bars denote 95% confidence intervals and letters above bars denote significant mean differences from a two‐tailed *t*‐test (*α* = 0.05).

In contrast to growth, we failed to detect appreciably different responses between the xerophytic oak/hickory and mesophyte groups for mortality (Figure 4). Despite the severity of the 2012 drought in terms of climate abnormality, terrestrial carbon uptake, and crop yield (Mallya et al. 2013; Yi et al. 2017), we found only modest impacts on forest die‐off. Across all FIA plots and species, the acceleration of stem loss ($∆m_{i}$; Equation 6) was less than 2%. Nevertheless, within the different functional groups, important differences between species did emerge.

Unlike growth, relative mortality sensitivity was highly diverse across the individual oak/hickory and mesophytic species (coefficient of variation = 6.69 and 8.01 for oak/hickory and mesophytes, respectively; Figure 4a). Although some oak/hickory species were similarly tolerant in terms of growth and survival (e.g., shagbark hickory and pignut hickory; Figures 3a and 4a), others (e.g., black hickory and black oak) experienced some of the greatest relative stem losses. Likewise, many mesophytes, which are putatively considered sensitive to drought, survived in greater numbers than the majority of their xerophytic neighbors. For example, red maple, American beech, and black gum had lower relative mortality sensitivity metric values than every oak/hickory species but shagbark hickory and pignut hickory (Figure 4a). Overall, we found that drought‐driven stem loss rates between the xerophytic oak/hickory and mesophytic groups were statistically indistinguishable (Figure 4b). The iterative stem loss comparisons between individual species pairs that informed the relative mortality sensitivity metric ($m_{r\_\mathrm{ij}}$; Equation 7) are reported in the (Figure S7).

**FIGURE 5:**
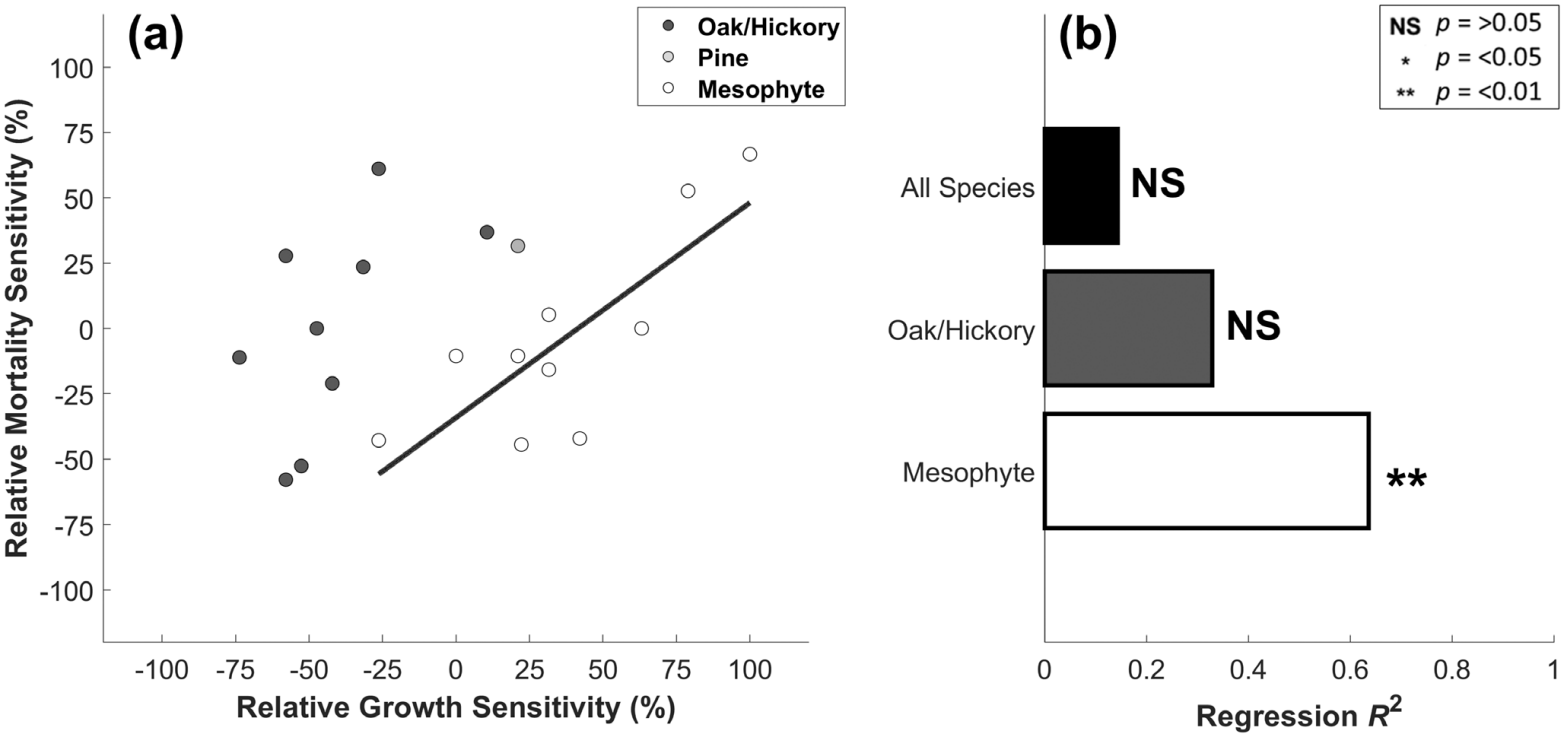
Relationship between relative growth sensitivity and relative mortality sensitivity. Panel (b) are *R* ^2^ estimates from linear regression analyses evaluated across all species and functional groups. Lines in panel (a) are best fit from significant linear regressions (*α* = 0.05).

Because relative growth sensitivity among functional groups was highly clustered (Figure 3a) but relative mortality sensitivity was not (Figure 4a), growth sensitivity across all species was weakly related to mortality risk following severe drought. When all species were considered, we found no significant relationship between these two drought‐tolerant metrics (Figure 5). Likewise, there was no significant relationship among the xerophytic oak and hickory species. For mesophytes, however, we found a positive relationship between growth sensitivity and survival sensitivity (Figure 5b). We additionally repeated this analysis among a larger subset of trees (i.e., trees with a DBH greater than 20 cm), but it did not meaningfully affect our results (Figure S8).

## 4 Discussion

By evaluating tree growth and mortality patterns during a severe drought event, we generate a novel perspective on the potential carbon consequences of forest compositional shifts between mesophytes and xerophytes. When comparing co‐located species, our analyses confirmed prior expectations that mesophytes are more sensitive to drought than xerophytes in terms of growth (Niinemets and Valladares 2006; Brzostek et al. 2014; Hu et al. 2017), but not mortality. We additionally revealed that growth sensitivity to water deficits was broadly decoupled from mortality risk across a diverse assemblage of tree species. These findings suggest that, during drought, declining xerophytic abundance has a larger impact on growth than on survival. However, xerophytes and mesophytes exhibited strong functional differences regarding the relationship between these two important drought‐tolerant metrics. While there was no relationship between relative growth and relative mortality sensitivities across all species, they were positively coordinated within the mesophyte group (Figure 5). Therefore, in regions where mesophyte establishment is being favored (Bhatta and Vetaas 2016; Spînu et al. 2020; Alexander et al. 2021), forest stands with limited growth sensitivity are likely to be the most tolerant to severe drought events moving forward.

### 4.1 Growth–Mortality Coordination Differs Among Xerophytes Versus Mesophytes During Severe Drought

Importantly, our results demonstrate that characterizing drought sensitivity depends on whether growth or mortality is the metric used to define its impact. Many of our analyses supported the perspective that the oak and hickory species in our study exhibit xerophytic characteristics (Fralish 2004; Alexander et al. 2021); they were most abundant in arid landscapes (Figure S4) and were the least sensitive species to drought in terms of growth (Figure 3). Additionally, oak and hickories possess many traits that are well‐adapted to water scarcity, including low specific leaf area, deep roots which can access stable soil moisture pools, and desiccation‐resistant leaves (Abrams 1990; Valladares and Niinemets 2008; Alexander et al. 2021). Despite these adaptations, their growth insensitivity to water deficits is also enabled by their propensity to operate with small hydraulic safety margins (Kannenberg, Novick, et al. 2019; Benson et al. 2022; Novick et al. 2022), which is a hydraulic strategy indicative of acute mortality risk (Delzon and Cochard 2014; Anderegg et al. 2016). Thus, we expected that their greater relative growth during severe drought would be accompanied by elevated stem loss (Fan et al. 2012; Haavik et al. 2015; Radcliffe et al. 2021). Instead, our findings suggest that even if oak and hickories sustained their growth by incurring substantial embolism, it did not contribute to a disproportionate risk for die‐off (Choat et al. 2012).

If xerophytic oak and hickories can tolerate appreciable xylem embolism, this could explain the lack of coordination between drought‐driven growth and mortality we observed across species and functional groups. There is overwhelming evidence that oaks and hickories readily allow substantial plant water potential declines yet develop embolism‐vulnerable xylem (Roman et al. 2015; Gu et al. 2015; Kannenberg, Novick, et al. 2019; Benson et al. 2022; Novick et al. 2022). However, oak and hickory species also rely on shallower sapwood depths to conduct water than mesophytes and thus have greater capacity to recover from hydraulic dysfunction via new growth (Brodribb et al. 2010; Tao et al. 2024). Such a strategy encompasses substantial risk, as evidenced by the drought legacy effects that are especially pronounced for oak and hickory species (Kannenberg, Novick, Alexander, et al. 2019). On the other hand, it may also be a competitively advantageous strategy to establish dominance over faster‐growing, but drought‐sensitive mesophytes (Pederson et al. 2015).

In contrast to the xerophytic oak/hickory group, mesophytes exhibited a strong positive coordination between relative growth and relative mortality sensitivities (Figure 5). Mesophytes' investment in traits that capitalize on moisture abundance to overtop their neighbors, including a high relative allocation to leaf biomass that enhances light interception and productivity (Wright et al. 2010; Alexander et al. 2021), makes them sensitive to water deficits (Jump et al. 2017), but can also decrease the time to carbon starvation (McDowell et al. 2008; Gentine et al. 2016). Though mesophytes tend to avoid desiccation during drought by more strongly down‐regulating their water use and carbon uptake than xerophytes (Roman et al. 2015; Alexander et al. 2021; Novick et al. 2022), prolonged stomatal closure may sufficiently deplete their carbon reserves to an extent that sets them on a trajectory towards die‐off (McDowell et al. 2008, 2011). Because declining growth is often an indicator preceding mortality (Liu et al. 2019; DeSoto et al. 2020) risk of carbon starvation could explain why the mesophytic species in our study that experienced greater growth reductions also experienced greater mortality.

Because forest compositional shifts over the last century have largely been attributed to climate change and management (Bhatta and Vetaas 2016; Spînu et al. 2020; Trugman et al. 2020; Alexander et al. 2021), it is important to consider the extent to which our functional groups and study species may be asymmetrically impacted by these anthropogenic drivers. For example, in our study region, fire suppression and wet climate conditions have inhibited xerophytic oak‐hickory regeneration (McEwan et al. 2011; Pederson et al. 2015), such that the mesophytic species that are replacing them are skewed towards smaller tree sizes and occur more frequently in subcanopy positions (Fei et al. 2011; McEwan et al. 2011). While drought impacts can vary substantially across canopy strata (Orwig and Abrams 1997; Bennett et al. 2015; Au et al. 2022), we found little evidence that these differences affected our results. When the relationship between relative growth and relative mortality sensitivities was evaluated among a subset of larger trees (trees with a DBH greater than 20 cm), our main findings did not substantially change (Figure S8). Within the mesophyte group, however, structural influences were more nuanced. Tree size had a disproportionate impact on mortality for sassafras (relative mortality sensitivity was 5.26% and 80.0% at the > 12.7 cm and > 20 cm threshold, respectively), though growth sensitivity remained positively coordinated with survival sensitivity among the other mesophytes (*R* ^2^ = 0.778, *p* = < 0.01; Figure S8b). Regardless, the lack of a relationship between these metrics across all species from the larger tree subset (*R* ^2^ = 0.125, *p* = 0.137; Figure S8b) suggests that structural differences in our study region do not explain the different drought‐driven growth and mortality relationships observed between xerophytes versus mesophytes.

### 4.2 Advantages and Limitations of Co‐Located Methodologies for Evaluating Drought‐Responses

To evaluate drought‐driven growth and mortality dynamics across species and functional groups from inventory data, we developed a novel approach that relied on a carefully designed suite of algorithms to compare relative responses between co‐located species. Though this strategy differs from many commonly used statistical approaches, it also provides many advantages. For example, inventory analyses routinely account for topographic factors (Kromroy et al. 2008; Klos et al. 2009; Thompson 2009; Venturas et al. 2021) but rarely consider differences among species‐specific geographic distributions. In our study, the ranges of the 20 highly abundant study species were considerably variable (Figure S2). Even if these species shared important topographic similarities (e.g., hillslope position, latitude, climate, etc.), they were growing in spatially distinct locations and may have been subject to different disturbance legacies. By comparing responses only within their overlapping regions, these types of topographically independent influences are minimized.

This approach can additionally account for the overwhelmingly numerous and complex suite of environmental factors that determine drought responses but are often challenging to directly assess. While the simple plot‐level characteristics that accompany inventory data sets are regularly included in statistical models (Kromroy et al. 2008; Klos et al. 2009; Thompson 2009; Venturas et al. 2021), many environmental factors which are known to influence drought sensitivity are not because they are either unavailable, temporally mismatched, and/or difficult to characterize with high fidelity. Such factors may include depth to bedrock, nutrient environments, soil hydraulic properties, and beyond (Quesada et al. 2009). By comparing relative drought responses only to their neighbors, the influence from environmental factors that are difficult to evaluate statistically is also minimized.

The generation of standardized relative drought‐response metrics also makes this approach primed to answer pressing ecophysiological questions that are beyond the scope of this study. By minimizing the influence of landscape factors from landscape observations, these methods leverage data rich resources to establish a relative drought sensitivity baseline that is defined at the species level. Together with the accumulating wealth of knowledge in other networks like TRY (Kattge et al. 2011), XFT (Choat et al. 2012), and PSInet (Restrepo‐Acevedo et al. 2024), this information can be used to advance our understanding of the extent to which functional plant traits and/or physiological strategies confer drought tolerance.

Despite these advantages, it is also important to acknowledge the limitations of our approach. Quantifying drought responses (especially mortality) can be sensitive to small stem counts (Sheil et al. 1995; Zhou et al. 2021). Because inventory programs like FIA sample plots that are relatively modest in size (i.e., 42 m^2^), it is common to have just one or two individuals per plot when filtered by discrete species. To account for this, we focused on species with a high number of observations in the dataset and applied a tessellation scheme to group plots into larger spatial units. However, this means that our approach was only able to evaluate drought responses among a relatively limited number of species, albeit ones that disproportionately contribute to ecosystem function (i.e., species that accounted for ~80% of all regional FIA observations; Table 1). Moreover, while the methodology was designed to minimize the influence of a wide range of environmental factors, our approach to dividing and aggregating plots may not fully resolve the impact of fine‐scale variation in environmental conditions, which were assumed to be homogeneous within our unit of analysis (i.e., the ~875 km^2^ hexagons; Figure 1c).

Another potential limitation of our methodology is that it cannot account for influences arising from the timing of drought. The 2012 drought event that informed our results occurred relatively early in the growing season (Mallya et al. 2013; Yi et al. 2017) and it is possible that early season droughts can impose asymmetrical responses across our study species. For example, while most of the mesophytic species in the central hardwoods have a diffuse‐porous wood anatomy, oak and hickory species have ring‐ and semi‐ring‐porous wood, respectively (Au and Maxwell 2022). Thus, oaks and hickories construct large xylem elements early in the growing season that are efficient at transporting water but are also highly vulnerable to drought‐driven embolism (Christman et al. 2012). However, the different relative densities of large early wood xylem vessels in the respective ring‐ and semi‐ring‐porous wood of oak and hickory species may mean that the 2012 drought had a disproportionate impact across the genera that comprised our xerophyte group (Table 1). To further understand how composition changes between xerophytes and mesophytes alter the relationship between drought‐driven growth and mortality, future studies should prioritize investigating the extent to which drought timing may influence these dynamics (D'Orangeville et al. 2018).

### 4.3 Implications for Eastern United States Forest Management and Beyond

Projecting how oak‐hickory decline will alter the ecosystem services of eastern United States forests requires insights into the degree that forest compositional shifts may alter growth and mortality patterns. By comparing drought‐driven growth responses among co‐located species, our analyses reaffirmed long‐standing concerns that the ongoing decline of xerophytic oak/hickory species will make regional forest productivity increasingly sensitive to drought (Brzostek et al. 2014; Coble et al. 2017; Iverson et al. 2018; Au et al. 2020; Novick et al. 2022). However, mortality is also an important component of terrestrial carbon balance (van der Molen et al. 2011; Xu et al. 2012) and our analyses additionally revealed that xerophytic oak/hickory species are just as likely to perish from severe water deficits as the mesophytes that are replacing them. Nevertheless, total stem loss during the exceptional 2012 drought was relatively low (less than 2% acceleration on average across species), suggesting that regional forest productivity during severe droughts moving forward is more likely to be diminished by reduced growth, as opposed to carbon losses via mortality (Maxwell et al. 2024). In that regard, our results suggest the conservation and regeneration of oak‐hickory forests is vital to sustain the current carbon mitigation potential of this highly productive terrestrial carbon sink.

Though our study leveraged a drought event in the eastern United States to evaluate how growth and mortality dynamics differ between xerophytes versus mesophytes, our findings have farther‐reaching implications. Forest compositional shifts are occurring in many regions of the globe, often by similar mechanisms to those in our study region. For example, xerophytic oak decline over the last century has additionally been reported in Europe, Asia, and northern Africa, and land management (especially fire suppression) has often been implicated as the leading driver (Haavik et al. 2015; Bhatta and Vetaas 2016; Spînu et al. 2020; Gosling et al. 2024). Broadly, our findings suggest that as these forests continue to decline in xerophytic abundance, their drought‐driven mortality risk may be relatively unchanged, but their growth will become increasingly sensitive to water deficits. Given the predicted climate changes for the future (IPCC 2023), management efforts to slow ongoing compositional shifts will be decisive in determining the carbon balance fate of these forest systems.

## Author Contributions

**Michael C. Benson:** conceptualization, formal analysis, funding acquisition, methodology, writing – original draft, writing – review and editing. **Taehee Hwang:** formal analysis, methodology, writing – review and editing. **Justin T. Maxwell:** conceptualization, writing – review and editing. **Richard P. Phillips:** conceptualization, writing – review and editing. **Kimberly A. Novick:** conceptualization, funding acquisition, methodology, writing – review and editing.

## Conflicts of Interest

The authors declare no conflicts of interest.

## Supporting information


Appendix S1.
**Figure S1.** Example of the spatial smoothing approach described in Section 2.1 in the main text. The unit of analysis for this study was defined as the individuals in hexagon plus the sum of those in their surrounding hexagon. This smoothing scheme was applied to all species and hexagons.
**Figure S2.** FIA live stem counts (*n*) in each hexagon during the initial pre‐drought period (from 2000 to 2005). Map lines delineate study areas and do not necessarily depict accepted national boundaries.
**Figure S3.** Distribution of xerophytic oak/hickory species (panel (a)) and mesophytic species (panel (b)) composition in each hexagon during the initial pre‐drought period (from 2000 to 2005). Composition refers to the percentage of individuals within functional groups present in the FIA survey (Table S1).
**Figure S4.** Species‐specific historical climate conditions across the study area. Panel (a) is mean aridity wetness index (Figure 2b) weighted by live stem counts of individual species with error bars denoting standard errors. Panel (b) is mean aridity‐wetness index weighted by live stem counts across functional groups. Aridity wetness index denotes the ratio of mean annual precipitation to mean annual evapotranspiration at 30 arc‐second spatial resolution from 1970 to 2000 (Trabucco & Zomer, 2019). Error bars in panel (b) are 95% confident intervals and letters above bars indicate significant differences from a weighted two‐tailed *t*‐test (*α* = 0.05). Oak and hickories are more abundant in the drier landscapes within our study region, confirming their widely recognized xerophytic nature.
**Figure S5.** An assessment of the extent that 2012 drought responses were sensitive to the historical climate conditions across the distinct landscape positions of mesophytes versus xerophytes. Panel (a) is species‐specific Pearson correlation coefficients (*r*) between growth response following drought (∆RGR; Equation 2) and mean aridity‐wetness index from 1970 to 2000 (Trabucco and Zomer 2018) in the hexagons across their range (i.e., Figure S2). Panel (c) is species‐specific Pearson correlation coefficients (*r*) between mortality response following drought (∆m; Equation 6) and aridity‐wetness index from 1970 to 2000 in the hexagons across their range (i.e., Figure S2). Panels (b) and (d) are mean differences across functional groups for growth and mortality responses, respectively. Error bars denote 95% confidence intervals around parameter estimates. Letters above bars in panels (b) and (d) denote significant differences from a two‐tail *z‐*test (*α* = 0.05). Overall, the confounding influence of species‐specific ranges and their historical climate envelopes promoted diverse responses. These results motivate the need to minimize environmental covariance when determining species‐level droughtresponses, which we do by evaluating relative growth and mortality *only* among co‐located individuals (Sections 2.3 and 2.4 in the main text).
**Figure S6.** The iterative growth comparisons ($g_{r}$) between individual species pairs at the hexagon‐level (Section 2.3 in the main text). Panels are pairwise difference comparisons between corrected relative growth rate of specific species (title) and those present in the same hexagons (*x*‐axis). Green arrows denote the title species had greater growth following drought, a red arrow denotes the title species had reduced growth following drought, and ‘×’ denotes both species experienced similar growth responses. Significant differences in responses were determined by one‐sample *t*‐test at the *α* = 0.05. Species codes are listed in Table 1 in the main text.
**Figure S7.** The iterative stem loss comparisons ($m_{r}$) between individual species pairs at the hexagon‐level (Section 2.4 in the main text). Panels are pairwise difference comparisons between corrected stem loss rates of a specific species (title) and those present in the same hexagons (*x*‐axis). Green arrows denote the title species had lower stem loss following drought, a red arrow denotes the title species had greater stem loss following drought, and ‘×’ denotes both species experienced similar stem loss responses. Significant differences in responses were determined by one‐sample *t*‐test at the *α* = 0.05. Species codes are listed in Table 1 in the main text.
**Figure S8.** Relationship between relative growth sensitivity (%) and relative mortality sensitivity (%) among a lager tree subset, defined by species with a diameter at breast height greater than 20 cm. Panel (b) are *R* ^2^ estimates from linear regression analyses evaluated across all species and functional groups (Table 1 in the main text). Lines in panel (a) are best fit from significant linear regressions (*α* = 0.05).
**Table S1.** FIA live stem counts (*n*) in each hexagon during the initial pre‐drought period (from 2000 to 2005). Latitude and Longitude columns are the center coordinates of each hexagon (Hex ID). Species codes are listed in Table 1 in the main text.

## Data Availability

Forest Inventory datasets used in this work are publicly available from the US Department of Agriculture Forest Service, Forest Inventory and Analysis Database and were accessed at https://research.fs.usda.gov/products/dataandtools/fia‐datamart (version 2.0.1) by downloading National Forest Inventory (NFI) datasets for the US states of Indiana, Illinois, Kentucky and Missouri. Global Aridity‐Wetness index data are publicly available and were accessed at: https://figshare.com/articles/dataset/Global_Aridity_Index_and_Potential_Evapotranspiration_ET0_Climate_Database_v2/7504448/3 (version 2). Processed data and code that support the findings in this work, including species‐specific relative growth responses (i.e., $g_{r\_\mathrm{ij}}$), relative mortality responses (i.e., $m_{r\_\mathrm{ij}})$, relative growth sensitivities, and relative mortality sensitivities, are openly available in Dryad at: https://doi.org/10.5061/dryad.280gb5n2b.

## References

[gcb70260-bib-0001] Abrams, M. D. 1990. “Adaptations and Responses to Drought in *Quercus* Species of North America.” Tree Physiology 7: 227–238. 14972920 10.1093/treephys/7.1-2-3-4.227

[gcb70260-bib-0002] Abrams, M. D. 2003. “Where Has All the White Oak Gone?” Bioscience 53, no. 10: 927–939.

[gcb70260-bib-0003] Adler, P. B. , R. Salguero‐Gómez , A. Compagnoni , et al. 2014. “Functional Traits Explain Variation in Plant Life History Strategies.” Proceedings of the National Academy of Sciences 111, no. 2: 740–745. 10.1073/pnas.1315179111 PMC3896207 24379395

[gcb70260-bib-0004] Alexander, H. D. , C. Siegert , J. S. Brewer , et al. 2021. “Mesophication of Oak Landscapes: Evidence, Knowledge Gaps, and Future Research.” Bioscience 71, no. 5: 531–542.

[gcb70260-bib-0005] Allen, C. D. , and D. D. Breshears . 1998. “Drought‐Induced Shift of a Forest–Woodland Ecotone: Rapid Landscape Response to Climate Variation.” Proceedings of the National Academy of Sciences 95, no. 25: 14839–14842. 10.1073/pnas.95.25.14839 PMC24536 9843976

[gcb70260-bib-0006] Anderegg, W. R. , T. Klein , M. Bartlett , et al. 2016. “Meta‐Analysis Reveals That Hydraulic Traits Explain Cross‐Species Patterns of Drought‐Induced Tree Mortality Across the Globe.” Proceedings of the National Academy of Sciences of the United States of America 113, no. 18: 5024–5029. 27091965 10.1073/pnas.1525678113 PMC4983847

[gcb70260-bib-0007] Arthur, M. A. , B. A. Blankenship , A. Schörgendorfer , D. L. Loftis , and H. D. Alexander . 2015. “Changes in Stand Structure and Tree Vigor With Repeated Prescribed Fire in an Appalachian Hardwood Forest.” Forest Ecology and Management 340: 46–61.

[gcb70260-bib-0008] Au, T. F. , and J. T. Maxwell . 2022. “Drought Sensitivity and Resilience of Oak–Hickory Stands in the Eastern United States.” Forests 13, no. 3: 389.

[gcb70260-bib-0009] Au, T. F. , J. T. Maxwell , K. A. Novick , et al. 2020. “Demographic Shifts in Eastern US Forests Increase the Impact of Late‐Season Drought on Forest Growth.” Ecography 43, no. 10: 1475–1486.

[gcb70260-bib-0010] Au, T. F. , J. T. Maxwell , S. M. Robeson , et al. 2022. “Younger Trees in the Upper Canopy Are More Sensitive but Also More Resilient to Drought.” Nature Climate Change 12, no. 12: 1168–1174.

[gcb70260-bib-0011] Augusto, L. , R. Borelle , A. Boča , et al. 2025. “Widespread Slow Growth of Acquisitive Tree Species.” Nature 640: 1–7. 10.1038/s41586-025-08692-x 40108455

[gcb70260-bib-0012] Bechtold, W. A. , and P. L. Patterson . 2005. The Enhanced Forest Inventory and Analysis Program—National Sampling Design and Estimation Procedures (No. 80). USDA Forest Service, Southern Research Station.

[gcb70260-bib-0013] Bennett, A. C. , N. G. McDowell , C. D. Allen , and K. J. Anderson‐Teixeira . 2015. “Larger Trees Suffer Most During Drought in Forests Worldwide.” Nature Plants 1, no. 10: 1–5. 10.1038/nplants.2015.139 27251391

[gcb70260-bib-0014] Benson, M. C. , C. F. Miniat , A. C. Oishi , et al. 2022. “The Xylem of Anisohydric *Quercus alba* L. Is More Vulnerable to Embolism Than Isohydric Codominants.” Plant, Cell & Environment 45, no. 2: 329–346. 10.1111/pce.14244 34902165

[gcb70260-bib-0015] Bhatta, K. P. , and O. R. Vetaas . 2016. “Does Tree Canopy Closure Moderate the Effect of Climate Warming on Plant Species Composition of Temperate Himalayan Oak Forest?” Journal of Vegetation Science 27, no. 5: 948–957.

[gcb70260-bib-0016] Brandt, L. , H. He , L. Iverson , et al. 2014. “Central Hardwoods Ecosystem Vulnerability Assessment and Synthesis: A Report From the Central Hardwoods Climate Change Response Framework Project.” General Technical Report NRS‐124. Newtown Square, PA: US Department of Agriculture, Forest Service, Northern Research Station. 124, 254.

[gcb70260-bib-0017] Brodribb, T. J. , D. J. Bowman , S. Nichols , S. Delzon , and R. Burlett . 2010. “Xylem Function and Growth Rate Interact to Determine Recovery Rates After Exposure to Extreme Water Deficit.” New Phytologist 188, no. 2: 533–542. 20673281 10.1111/j.1469-8137.2010.03393.x

[gcb70260-bib-0018] Brzostek, E. R. , D. Dragoni , H. P. Schmid , et al. 2014. “Chronic Water Stress Reduces Tree Growth and the Carbon Sink of Deciduous Hardwood Forests.” Global Change Biology 20, no. 8: 2531–2539. 24421179 10.1111/gcb.12528

[gcb70260-bib-0019] Buckley, T. N. , L. Sack , and G. D. Farquhar . 2017. “Optimal Plant Water Economy.” Plant, Cell & Environment 40, no. 6: 881–896. 10.1111/pce.12823 27644069

[gcb70260-bib-0020] Cavender‐Bares, J. 2016. “Diversity, Distribution and Ecosystem Services of the North American Oaks.” International Oaks 27, no. 27: 37–48.

[gcb70260-bib-0021] Choat, B. , S. Jansen , T. J. Brodribb , et al. 2012. “Global Convergence in the Vulnerability of Forests to Drought.” Nature 491, no. 7426: 752–755. 23172141 10.1038/nature11688

[gcb70260-bib-0022] Christman, M. A. , J. S. Sperry , and D. D. Smith . 2012. “Rare Pits, Large Vessels and Extreme Vulnerability to Cavitation in a Ring‐Porous Tree Species.” New Phytologist 193, no. 3: 713–720. 22150784 10.1111/j.1469-8137.2011.03984.x

[gcb70260-bib-0023] Coble, A. P. , M. A. Vadeboncoeur , Z. C. Berry , et al. 2017. “Are Northeastern US Forests Vulnerable to Extreme Drought?” Ecological Processes 6, no. 1: 1–13.

[gcb70260-bib-0024] Delzon, S. , and H. Cochard . 2014. “Recent Advances in Tree Hydraulics Highlight the Ecological Significance of the Hydraulic Safety Margin.” New Phytologist 203, no. 2: 355–358. 24661229 10.1111/nph.12798

[gcb70260-bib-0025] Denham, S. O. , A. C. Oishi , C. F. Miniat , et al. 2021. “Eastern US Deciduous Tree Species Respond Dissimilarly to Declining Soil Moisture but Similarly to Rising Evaporative Demand.” Tree Physiology 41, no. 6: 944–959. 33185239 10.1093/treephys/tpaa153

[gcb70260-bib-0026] DeSoto, L. , M. Cailleret , F. Sterck , et al. 2020. “Low Growth Resilience to Drought Is Related to Future Mortality Risk in Trees.” Nature Communications 11, no. 1: 545. 10.1038/s41467-020-14300-5 PMC6987235 31992718

[gcb70260-bib-0027] D'Orangeville, L. , J. Maxwell , D. Kneeshaw , et al. 2018. “Drought Timing and Local Climate Determine the Sensitivity of Eastern Temperate Forests to Drought.” Global Change Biology 24, no. 6: 2339–2351. 29460369 10.1111/gcb.14096

[gcb70260-bib-0028] Fan, Z. , X. Fan , M. K. Crosby , et al. 2012. “Spatio‐Temporal Trends of Oak Decline and Mortality Under Periodic Regional Drought in the Ozark Highlands of Arkansas and Missouri.” Forests 3, no. 3: 614–631.

[gcb70260-bib-0029] FAO and UNEP . 2020. “The State of the World's Forests 2020.” Forests, Biodiversity and People Rome. 10.4060/ca8642en.

[gcb70260-bib-0030] Feeley, K. J. , S. J. Davies , R. Perez , S. P. Hubbell , and R. B. Foster . 2011. “Directional Changes in the Species Composition of a Tropical Forest.” Ecology 92, no. 4: 871–882. 21661550 10.1890/10-0724.1

[gcb70260-bib-0031] Fei, S. , J. M. Desprez , K. M. Potter , I. Jo , J. A. Knott , and C. M. Oswalt . 2017. “Divergence of Species Responses to Climate Change.” Science Advances 3, no. 5: e1603055. 28560343 10.1126/sciadv.1603055 PMC5435420

[gcb70260-bib-0032] Fei, S. , N. Kong , K. C. Steiner , W. K. Moser , and E. B. Steiner . 2011. “Change in Oak Abundance in the Eastern United States From 1980 to 2008.” Forest Ecology and Management 262, no. 8: 1370–1377.

[gcb70260-bib-0033] Fralish, J. S. 2004. “The Keystone Role of Oak and Hickory in the Central Hardwood Forest.” In Upland Oak Ecology Symposium: History, Current Conditions, and Sustainability: Fayetteville, Arkansas, vol. 73, 78. Southern Research Station.

[gcb70260-bib-0034] Gentine, P. , M. Guérin , M. Uriarte , N. G. McDowell , and W. T. Pockman . 2016. “An Allometry‐Based Model of the Survival Strategies of Hydraulic Failure and Carbon Starvation.” Ecohydrology 9, no. 3: 529–546.

[gcb70260-bib-0035] Gosling, R. H. , R. W. Jackson , M. Elliot , and C. P. Nichols . 2024. “Oak Declines: Reviewing the Evidence for Causes, Management Implications and Research Gaps.” Ecological Solutions and Evidence 5, no. 4: e12395.

[gcb70260-bib-0036] Gray, A. N. , T. J. Brandeis , J. D. Shaw , W. H. McWilliams , and P. D. Miles . 2012. “Forest Inventory and Analysis Database of the United States of America (FIA).” Biodiversity and Ecology 4: 225–231.

[gcb70260-bib-0037] Grubb, P. J. 1977. “The Maintenance of Species‐Richness in Plant Communities: The Importance of the Regeneration Niche.” Biological Reviews 52, no. 1: 107–145.

[gcb70260-bib-0038] Gu, L. , S. G. Pallardy , K. P. Hosman , and Y. Sun . 2015. “Predictors and Mechanisms of the Drought‐Influenced Mortality of Tree Species Along the Isohydric to Anisohydic Continuum in a Decade‐Long Study of a Central US Temperate Forest.” Biogeosciences Discussions 12, no. 2: 1285–1325.

[gcb70260-bib-0039] Haavik, L. J. , S. A. Billings , J. M. Guldin , and F. M. Stephen . 2015. “Emergent Insects, Pathogens and Drought Shape Changing Patterns in Oak Decline in North America and Europe.” Forest Ecology and Management 354: 190–205.

[gcb70260-bib-0040] Heath, L. S. , J. E. Smith , and R. A. Birdsey . 2002. “Carbon Trends in US Forestlands: A Context for the Role of Soils in Forest Carbon Sequestration.” In The Potential of US Forest Soils to Sequester Carbon and Mitigate the Greenhouse Effect, edited by R. A. Birdsey , 35–45. CRC Press.

[gcb70260-bib-0041] Holzmueller, E. J. , J. W. Groninger , and C. M. Ruffner . 2014. “Facilitating Oak and Hickory Regeneration in Mature Central Hardwood Forests.” Forests 5, no. 12: 3344–3351.

[gcb70260-bib-0042] Hu, H. , G. G. Wang , W. L. Bauerle , and R. J. Klos . 2017. “Drought Impact on Forest Regeneration in the Southeast USA.” Ecosphere 8, no. 4: e01772.

[gcb70260-bib-0043] IPCC . 2023. “Climate Change 2023: Synthesis Report.” In Contribution of Working Groups I, II and III to the Sixth Assessment Report of the Intergovernmental Panel on Climate Change, Core Writing Team, edited by H. Lee and J. Romero , 35–115. IPCC. 10.59327/IPCC/AR6-9789291691647.

[gcb70260-bib-0044] Iverson, L. R. , M. P. Peters , J. L. Bartig , et al. 2018. “Spatial Modeling and Inventories for Prioritizing Investment Into Oak‐Hickory Restoration.” Forest Ecology and Management 424: 355–366.

[gcb70260-bib-0045] Jo, I. , S. Fei , C. M. Oswalt , G. M. Domke , and R. P. Phillips . 2019. “Shifts in Dominant Tree Mycorrhizal Associations in Response to Anthropogenic Impacts.” Science Advances 5, no. 4: eaav6358. 30989116 10.1126/sciadv.aav6358 PMC6457943

[gcb70260-bib-0046] Jump, A. S. , P. Ruiz‐Benito , S. Greenwood , et al. 2017. “Structural Overshoot of Tree Growth With Climate Variability and the Global Spectrum of Drought‐Induced Forest Dieback.” Global Change Biology 23, no. 9: 3742–3757. 28135022 10.1111/gcb.13636

[gcb70260-bib-0047] Kannenberg, S. A. , K. A. Novick , M. R. Alexander , et al. 2019. “Linking Drought Legacy Effects Across Scales: From Leaves to Tree Rings to Ecosystems.” Global Change Biology 25, no. 9: 2978–2992. 31132225 10.1111/gcb.14710

[gcb70260-bib-0048] Kannenberg, S. A. , K. A. Novick , and R. P. Phillips . 2019. “Anisohydric Behavior Linked to Persistent Hydraulic Damage and Delayed Drought Recovery Across Seven North American Tree Species.” New Phytologist 222, no. 4: 1862–1872. 30664253 10.1111/nph.15699

[gcb70260-bib-0049] Kannenberg, S. A. , C. R. Schwalm , and W. R. Anderegg . 2020. “Ghosts of the Past: How Drought Legacy Effects Shape Forest Functioning and Carbon Cycling.” Ecology Letters 23, no. 5: 891–901. 32157766 10.1111/ele.13485

[gcb70260-bib-0050] Kattge, J. , S. Diaz , S. Lavorel , et al. 2011. “TRY–A Global Database of Plant Traits.” Global Change Biology 17, no. 9: 2905–2935.

[gcb70260-bib-0052] Klos, R. J. , G. G. Wang , W. L. Bauerle , and J. R. Rieck . 2009. “Drought Impact on Forest Growth and Mortality in the Southeast USA: An Analysis Using Forest Health and Monitoring Data.” Ecological Applications 19, no. 3: 699–708. 19425432 10.1890/08-0330.1

[gcb70260-bib-0053] Knapp, E. E. , A. A. Bernal , J. M. Kane , C. J. Fettig , and M. P. North . 2021. “Variable Thinning and Prescribed Fire Influence Tree Mortality and Growth During and After a Severe Drought.” Forest Ecology and Management 479: 118595.

[gcb70260-bib-0054] Kromroy, K. W. , J. Juzwik , P. Castillo , and M. H. Hansen . 2008. “Using Forest Service Forest Inventory and Analysis Data to Estimate Regional Oak Decline and Oak Mortality.” Northern Journal of Applied Forestry 25, no. 1: 17–24.

[gcb70260-bib-0055] Latham, R. E. 1992. “Co‐Occurring Tree Species Change Rank in Seedling Performance With Resources Varied Experimentally.” Ecology 73, no. 6: 2129–2144.

[gcb70260-bib-0056] Lévesque, M. , A. Rigling , H. Bugmann , P. Weber , and P. Brang . 2014. “Growth Response of Five Co‐Occurring Conifers to Drought Across a Wide Climatic Gradient in Central Europe.” Agricultural and Forest Meteorology 197: 1–12.

[gcb70260-bib-0057] Liu, Q. , C. Peng , R. Schneider , D. Cyr , N. G. McDowell , and D. Kneeshaw . 2023. “Drought‐Induced Increase in Tree Mortality and Corresponding Decrease in the Carbon Sink Capacity of Canada's Boreal Forests From 1970 to 2020.” Global Change Biology 29, no. 8: 2274–2285. 36704817 10.1111/gcb.16599

[gcb70260-bib-0058] Liu, Y. , M. Kumar , G. G. Katul , and A. Porporato . 2019. “Reduced Resilience as an Early Warning Signal of Forest Mortality.” Nature Climate Change 9, no. 11: 880–885.

[gcb70260-bib-0059] Lombardini, L. , and L. Rossi . 2019. “Ecophysiology of Plants in Dry Environments.” Dryland Ecohydrology: 71–100. https://link.springer.com/book/10.1007/978‐3‐030‐23269‐6.

[gcb70260-bib-0060] Mallya, G. , L. Zhao , X. C. Song , D. Niyogi , and R. S. Govindaraju . 2013. “2012 Midwest Drought in the United States.” Journal of Hydrologic Engineering 18, no. 7: 737–745.

[gcb70260-bib-0061] Maxwell, J. T. , T. F. Au , S. A. Kannenberg , et al. 2024. “Asymmetric Effects of Hydroclimate Extremes on Eastern US Tree Growth: Implications on Current Demographic Shifts and Climate Variability.” Global Change Biology 30, no. 8: e17474. 39162051 10.1111/gcb.17474

[gcb70260-bib-0062] Maxwell, J. T. , and G. L. Harley . 2017. “Increased Tree‐Ring Network Density Reveals More Precise Estimations of Sub‐Regional Hydroclimate Variability and Climate Dynamics in the Midwest, USA.” Climate Dynamics 49: 1479–1493.

[gcb70260-bib-0063] McDowell, N. , W. T. Pockman , C. D. Allen , et al. 2008. “Mechanisms of Plant Survival and Mortality During Drought: Why Do Some Plants Survive While Others Succumb to Drought?” New Phytologist 178, no. 4: 719–739. 18422905 10.1111/j.1469-8137.2008.02436.x

[gcb70260-bib-0064] McDowell, N. G. , D. J. Beerling , D. D. Breshears , R. A. Fisher , K. F. Raffa , and M. Stitt . 2011. “The Interdependence of Mechanisms Underlying Climate‐Driven Vegetation Mortality.” Trends in Ecology & Evolution 26, no. 10: 523–532. 21802765 10.1016/j.tree.2011.06.003

[gcb70260-bib-0065] McEwan, R. W. , J. M. Dyer , and N. Pederson . 2011. “Multiple Interacting Ecosystem Drivers: Toward an Encompassing Hypothesis of Oak Forest Dynamics Across Eastern North America.” Ecography 34, no. 2: 244–256.

[gcb70260-bib-0066] Meinzer, F. C. , D. R. Woodruff , D. M. Eissenstat , H. S. Lin , T. S. Adams , and K. A. McCulloh . 2013. “Above‐and Belowground Controls on Water Use by Trees of Different Wood Types in an Eastern US Deciduous Forest.” Tree Physiology 33, no. 4: 345–356. 23513033 10.1093/treephys/tpt012

[gcb70260-bib-0067] Niinemets, Ü. , and F. Valladares . 2006. “Tolerance to Shade, Drought, and Waterlogging of Temperate Northern Hemisphere Trees and Shrubs.” Ecological Monographs 76, no. 4: 521–547.

[gcb70260-bib-0068] Novick, K. , I. Jo , L. D'Orangeville , et al. 2022. “The Drought Response of Eastern US Oaks in the Context of Their Declining Abundance.” Bioscience 72, no. 4: 333–346.

[gcb70260-bib-0069] Orwig, D. A. , and M. D. Abrams . 1997. “Variation in Radial Growth Responses to Drought Among Species, Site, and Canopy Strata.” Trees 11, no. 8: 474–484.

[gcb70260-bib-0070] Pederson, N. , A. W. D'Amato , J. M. Dyer , et al. 2015. “Climate Remains an Important Driver of Post‐European Vegetation Change in the Eastern United States.” Global Change Biology 21, no. 6: 2105–2110. 25477234 10.1111/gcb.12779

[gcb70260-bib-0071] Pierce, A. R. , G. Parker , and K. Rabenold . 2006. “Forest Succession in an Oak‐Hickory Dominated Stand During a 40‐Year Period at the Ross Biological Reserve, Indiana.” Natural Areas Journal 26, no. 4: 351–359.

[gcb70260-bib-0072] Pugh, S. A. , A. M. Liebhold , and R. S. Morin . 2011. “Changes in Ash Tree Demography Associated With Emerald Ash Borer Invasion, Indicated by Regional Forest Inventory Data From the Great Lakes States.” Canadian Journal of Forest Research 41, no. 11: 2165–2175.

[gcb70260-bib-0073] Quesada, C. A. , J. Lloyd , M. Schwarz , et al. 2009. “Regional and Large‐Scale Patterns in Amazon Forest Structure and Function Are Mediated by Variations in Soil Physical and Chemical Properties.” Biogeosciences Discussions 6: 3993–4057.

[gcb70260-bib-0074] Radcliffe, D. C. , D. M. Hix , and S. N. Matthews . 2021. “Predisposing Factors' Effects on Mortality of Oak (*Quercus*) and Hickory (*Carya*) Species in Mature Forests Undergoing Mesophication in Appalachian Ohio.” Forest Ecosystems 8, no. 1: 1–14.

[gcb70260-bib-0075] Redmond, M. D. , N. S. Cobb , M. J. Clifford , and N. N. Barger . 2015. “Woodland Recovery Following Drought‐Induced Tree Mortality Across an Environmental Stress Gradient.” Global Change Biology 21, no. 10: 3685–3695. 26089027 10.1111/gcb.12976

[gcb70260-bib-0076] Reich, P. B. 2014. “The World‐Wide ‘Fast–Slow'plant Economics Spectrum: A Traits Manifesto.” Journal of Ecology 102, no. 2: 275–301.

[gcb70260-bib-0077] Restrepo‐Acevedo, A. M. , J. S. Guo , S. A. Kannenberg , et al. 2024. “PSInet: A New Global Water Potential Network.” Tree Physiology 44, no. 10: tpae110. 39190893 10.1093/treephys/tpae110 PMC11447379

[gcb70260-bib-0078] Roman, D. T. , K. A. Novick , E. R. Brzostek , D. Dragoni , F. Rahman , and R. P. Phillips . 2015. “The Role of Isohydric and Anisohydric Species in Determining Ecosystem‐Scale Response to Severe Drought.” Oecologia 179: 641–654. 26130023 10.1007/s00442-015-3380-9

[gcb70260-bib-0079] Sheil, D. , D. F. Burslem , and D. Alder . 1995. “The Interpretation and Misinterpretation of Mortality Rate Measures.” Journal of Ecology 83: 331–333.

[gcb70260-bib-0081] Spînu, A. P. , M. Niklasson , and E. Zin . 2020. “Mesophication in Temperate Europe: A Dendrochronological Reconstruction of Tree Succession and Fires in a Mixed Deciduous Stand in Białowieża Forest.” Ecology and Evolution 10, no. 2: 1029–1041. 32015862 10.1002/ece3.5966 PMC6988544

[gcb70260-bib-0082] Svoboda, M. , D. LeComte , M. Hayes , et al. 2002. “The Drought Monitor.” Bulletin of the American Meteorological Society 83, no. 8: 1181–1190.

[gcb70260-bib-0083] Tao, W. , J. He , N. G. Smith , et al. 2024. “Tree Growth Rate‐Mediated Trade‐Off Between Drought Resistance and Recovery in the Northern Hemisphere.” Proceedings of the Royal Society B: Biological Sciences 291, no. 2033: 20241427. 10.1098/rspb.2024.1427 PMC11521623 39471856

[gcb70260-bib-0084] Thompson, M. T. 2009. “Analysis of Conifer Mortality in Colorado Using Forest Inventory and Analysis's Annual Forest Inventory.” Western Journal of Applied Forestry 24, no. 4: 193–197.

[gcb70260-bib-0085] Tobler, W. R. 1969. “Geographical Filters and Their Inverses.” Geographical Analysis 1, no. 3: 234–253.

[gcb70260-bib-0086] Trabucco, A. , and R. J. Zomer . 2018. “Global Aridity Index and Potential Evapotranspiration (ET0) Climate Database v2.” CGIAR Consort Spat Inf, 10, m9. 10.1038/s41597-022-01493-1 PMC9287331 35840601

[gcb70260-bib-0087] Trugman, A. T. , L. D. Anderegg , J. D. Shaw , and W. R. Anderegg . 2020. “Trait Velocities Reveal That Mortality Has Driven Widespread Coordinated Shifts in Forest Hydraulic Trait Composition.” Proceedings of the National Academy of Sciences of the United States of America 117, no. 15: 8532–8538. 32229563 10.1073/pnas.1917521117 PMC7165451

[gcb70260-bib-0096] Valladares, F., and Ü. Niinemets. 2008. “Shade Tolerance, a Key Plant Feature of Complex Nature and Consequences.” Annual Review of Ecology, Evolution, and Systematics 39, no. 1: 237–257.

[gcb70260-bib-0088] van der Molen, M. K. , A. J. Dolman , P. Ciais , et al. 2011. “Drought and Ecosystem Carbon Cycling.” Agricultural and Forest Meteorology 151, no. 7: 765–773.

[gcb70260-bib-0089] Venturas, M. D. , H. N. Todd , A. T. Trugman , and W. R. Anderegg . 2021. “Understanding and Predicting Forest Mortality in the Western United States Using Long‐Term Forest Inventory Data and Modeled Hydraulic Damage.” New Phytologist 230, no. 5: 1896–1910. 33112415 10.1111/nph.17043

[gcb70260-bib-0090] Wright, S. J. , K. Kitajima , N. J. Kraft , et al. 2010. “Functional Traits and the Growth–Mortality Trade‐Off in Tropical Trees.” Ecology 91, no. 12: 3664–3674. 21302837 10.1890/09-2335.1

[gcb70260-bib-0091] Xiao, J. , Q. Zhuang , B. E. Law , et al. 2011. “Assessing Net Ecosystem Carbon Exchange of US Terrestrial Ecosystems by Integrating Eddy Covariance Flux Measurements and Satellite Observations.” Agricultural and Forest Meteorology 151, no. 1: 60–69.

[gcb70260-bib-0092] Xu, C. Y. , M. H. Turnbull , D. T. Tissue , et al. 2012. “Age‐Related Decline of Stand Biomass Accumulation Is Primarily due to Mortality and Not to Reduction in NPP Associated With Individual Tree Physiology, Tree Growth or Stand Structure in a *Quercus*‐Dominated Forest.” Journal of Ecology 100, no. 2: 428–440.

[gcb70260-bib-0093] Yi, K. , D. Dragoni , R. P. Phillips , D. T. Roman , and K. A. Novick . 2017. “Dynamics of Stem Water Uptake Among Isohydric and Anisohydric Species Experiencing a Severe Drought.” Tree Physiology 37, no. 10: 1379–1392. 28062727 10.1093/treephys/tpw126

[gcb70260-bib-0094] Zhao, C. , F. Brissette , J. Chen , and J. L. Martel . 2020. “Frequency Change of Future Extreme Summer Meteorological and Hydrological Droughts Over North America.” Journal of Hydrology 584: 124316.

[gcb70260-bib-0095] Zhou, X. , Q. Chen , R. P. Sharma , et al. 2021. “A Climate Sensitive Mixed‐Effects Diameter Class Mortality Model for Prince Rupprecht Larch (*Larix gmelinii* var. Principis‐Rupprechtii) in Northern China.” Forest Ecology and Management 491: 119091.

