# Supplementary material for: Growth–Mortality Coordination Differs Among Xerophytic Versus Mesophytic Tree Species During Severe Drought
Source: Glob Chang Biol. 2025 Jun 2;31(6):e70260. doi: 10.1111/gcb.70260 (PMC12127839; doi:10.1111/gcb.70260)
Supplement: Supplementary file 1 — Appendix S1. Figure S1. Example of the spatial smoothing approach described in Section 2.1 in the main text. The unit of analysis for this study was defined as the individuals in hexagon plus the sum of those in their surrounding hexagon. This smoothing scheme was applied to all species and hexagons. Figure S2. FIA live stem counts (n) in each hexagon during the initial pre‐drought period (from 2000 to 2005). Map lines delineate study areas and do not necessarily depict accepted national boundaries. Figure S3. Distribution of xerophytic oak/hickory species (panel (a)) and mesophytic species (panel (b)) composition in each hexagon during the initial pre‐drought period (from 2000 to 2005). Composition refers to the percentage of individuals within functional groups present in the FIA survey (Table S1). Figure S4. Species‐specific historical climate conditions across the study area. Panel (a) is mean aridity wetness index (Figure 2b) weighted by live stem counts of individual species with error bars denoting standard errors. Panel (b) is mean aridity‐wetness index weighted by live stem counts across functional groups. Aridity wetness index denotes the ratio of mean annual precipitation to mean annual evapotranspiration at 30 arc‐second spatial resolution from 1970 to 2000 (Trabucco & Zomer, 2019). Error bars in panel (b) are 95% confident intervals and letters above bars indicate significant differences from a weighted two‐tailed t‐test (α = 0.05). Oak and hickories are more abundant in the drier landscapes within our study region, confirming their widely recognized xerophytic nature. Figure S5. An assessment of the extent that 2012 drought responses were sensitive to the historical climate conditions across the distinct landscape positions of mesophytes versus xerophytes. Panel (a) is species‐specific Pearson correlation coefficients (r) between growth response following drought (∆RGR; Equation 2) and mean aridity‐wetness index from 1970 to 2000 (Trabucco and Zomer 20 [file GCB-31-e70260-s001.docx]

**Supporting Information for the manuscript:** ***Growth-mortality coordination differs among xerophytic vs. mesophytic tree species during severe drought***

**Figure S1.** Example of the spatial smoothing approach described in Section 2.1 in the main text. The unit of analysis for this study was defined as the individuals in hexagon plus the sum of those in their surrounding hexagon. This smoothing scheme was applied to all species and hexagons.


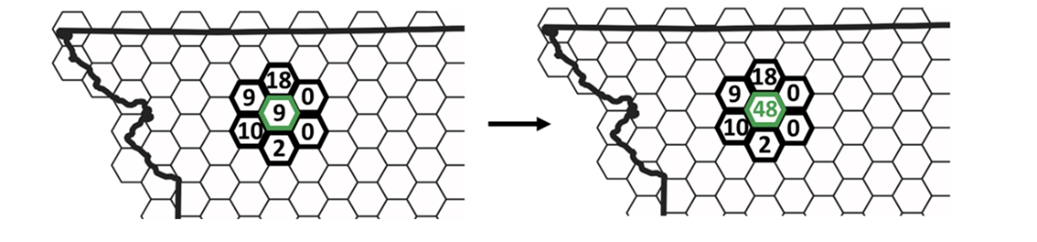


**Figure S2.** FIA live stem counts (n) in each hexagon during the initial pre-drought period (from 2000-2005). Map lines delineate study areas and do not necessarily depict accepted national boundaries.


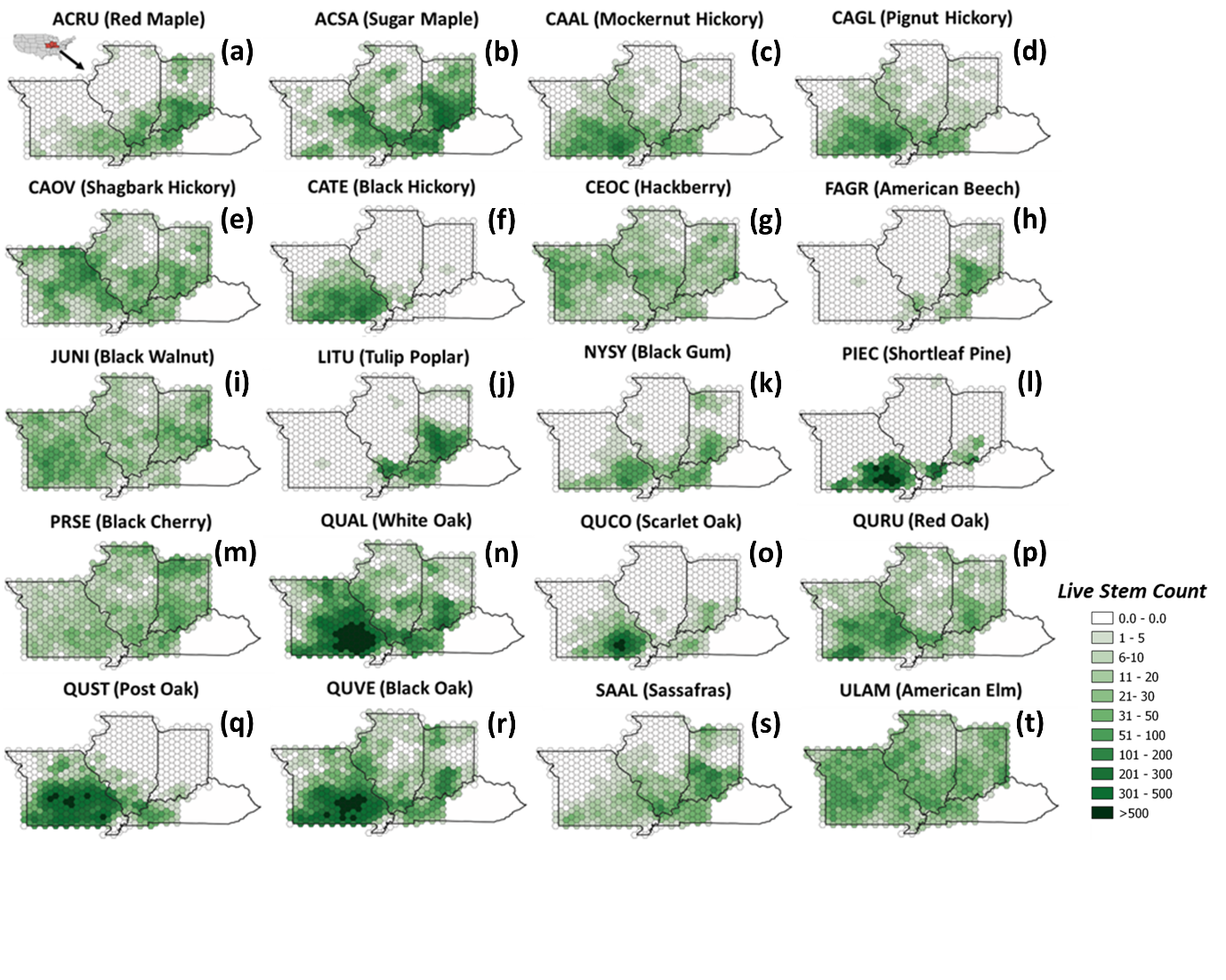


**Figure S3.** Distribution of xerophytic oak/hickory species (panel (a)) and mesophytic species (panel (b)) composition in each hexagon during the initial pre-drought period (from 2000 – 2005). Composition refers to the percentage of individuals within functional groups present in the FIA survey (Table S1).


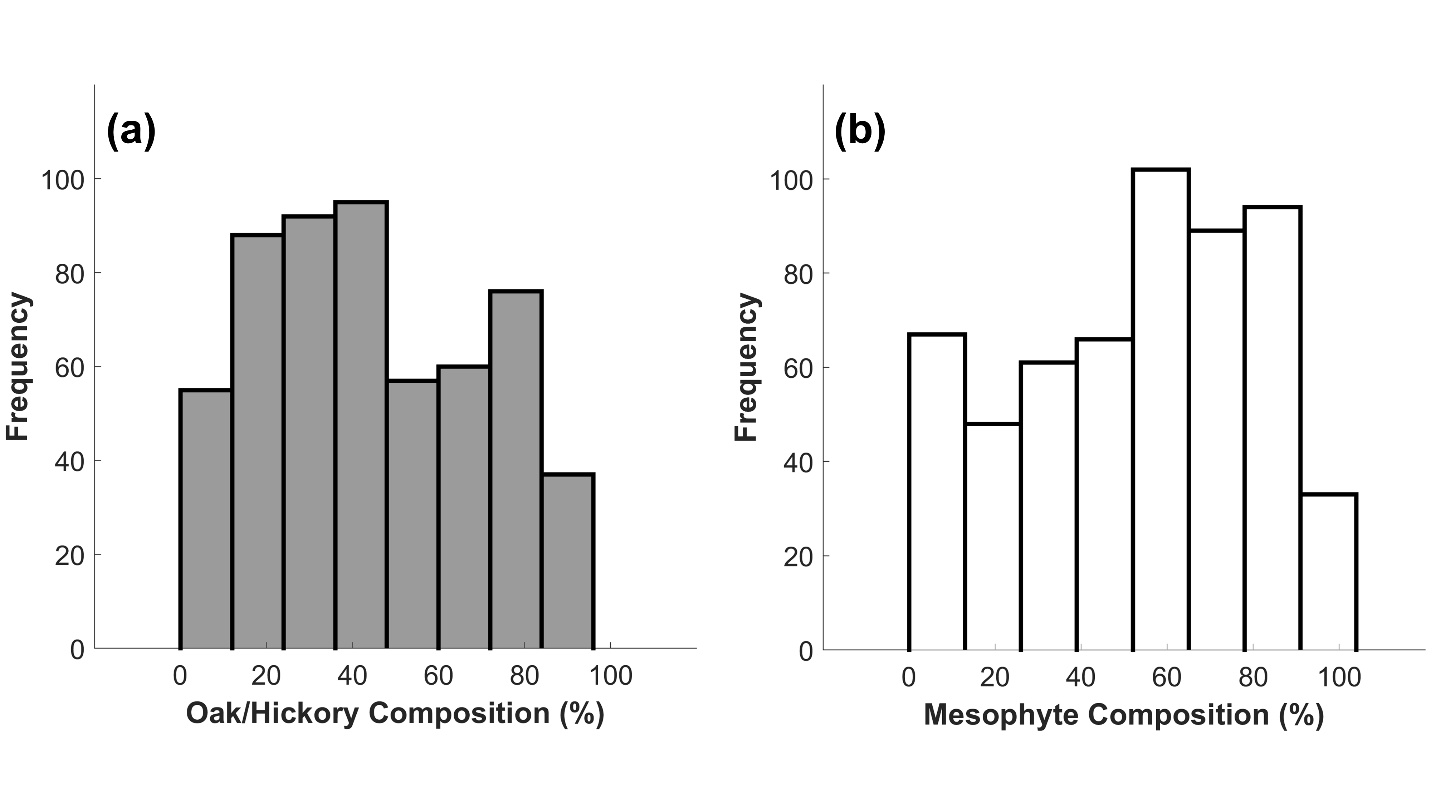


**Figure S4.** Species-specific historical climate conditions across the study area. Panel (a) is mean aridity wetness index (Fig. 2b) weighted by live stem counts of individual species with error bars denoting standard errors. Panel (b) is mean aridity-wetness index weighted by live stem counts across functional groups. Aridity wetness index denotes the ratio of mean annual precipitation to mean annual evapotranspiration at 30 arc-second spatial resolution from 1970 to 2000 (Trabucco & Zomer, 2019). Error bars in panel (b) are 95% confident intervals and letters above bars indicate significant differences from a weighted two-tailed *t*-test (*α* = 0.05). Oak and hickories are more abundant in the drier landscapes within our study region, confirming their widely recognized xerophytic nature.


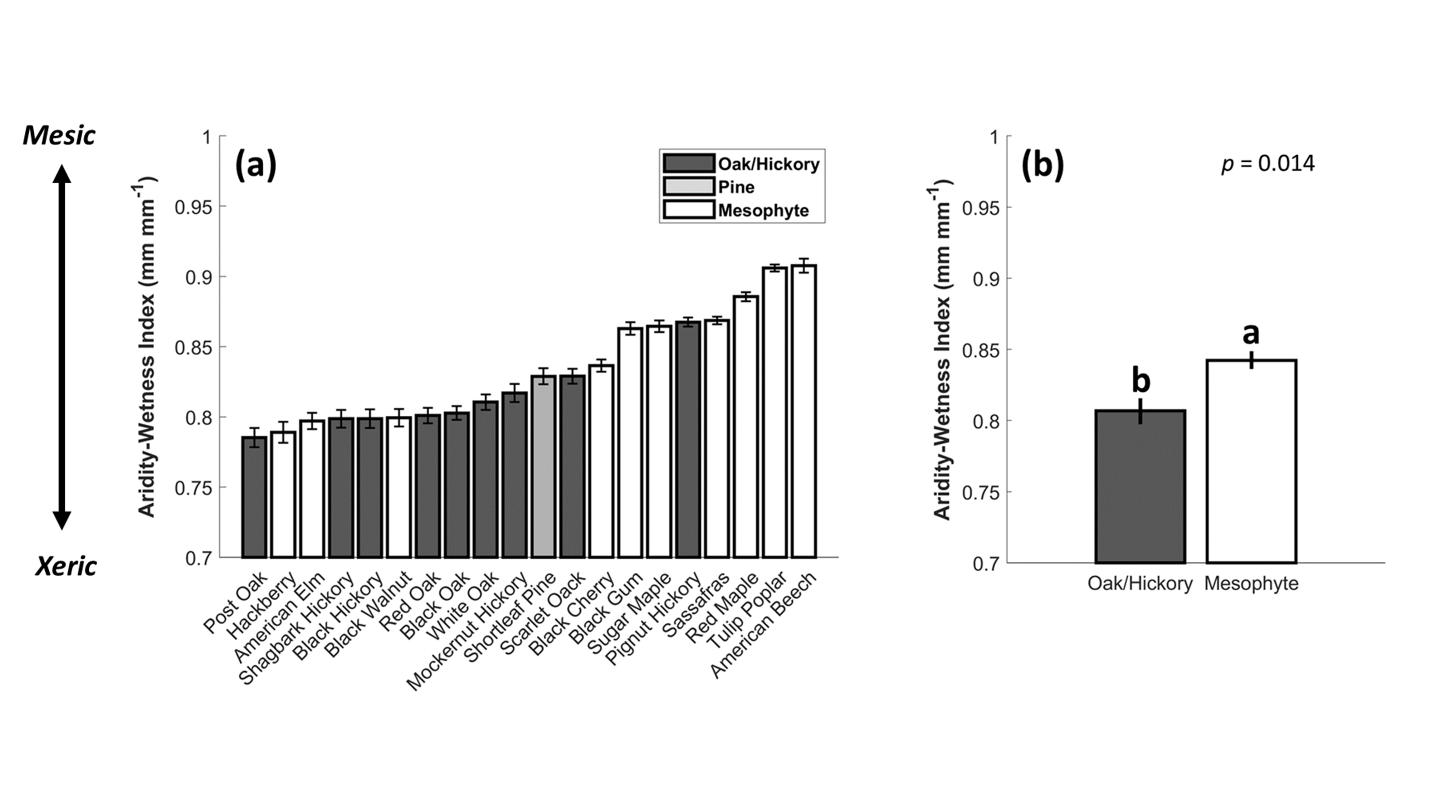


**Figure S5.** An assessment of the extent that 2012 drought responses were sensitive to the historical climate conditions across the distinct landscape positions of mesophytes vs. xerophytes. Panel (a) is species-specific Pearson correlation coefficients (*r*) between growth response following drought ($\Delta RGR$; Equation 2) and mean aridity-wetness index from 1970 to 2000 (Trabucco & Zomer, 2018) in the hexagons across their range (i.e., Fig. S2). Panel (c) is species-specific Pearson correlation coefficients (*r*) between mortality response following drought ($\Delta m$; Equation 6) and aridity-wetness index from 1970 to 2000 in the hexagons across their range (i.e., Fig. S2). Panels (b) and (d) are mean differences across functional groups for growth and mortality responses, respectively. Error bars denote 95% confidence intervals around parameter estimates. Letters above bars in panels (b) and (d) denote significant differences from a two-tail *z-*test (*α* = 0.05). Overall, the confounding influence of species-specific ranges and their historical climate envelopes promoted diverse responses. These results motivate the need to minimize environmental covariance when determining species-level droughtresponses, which we do by evaluating relative growth and mortality *only* among co-located individuals (Sections 2.3 and 2.4 in the main text).


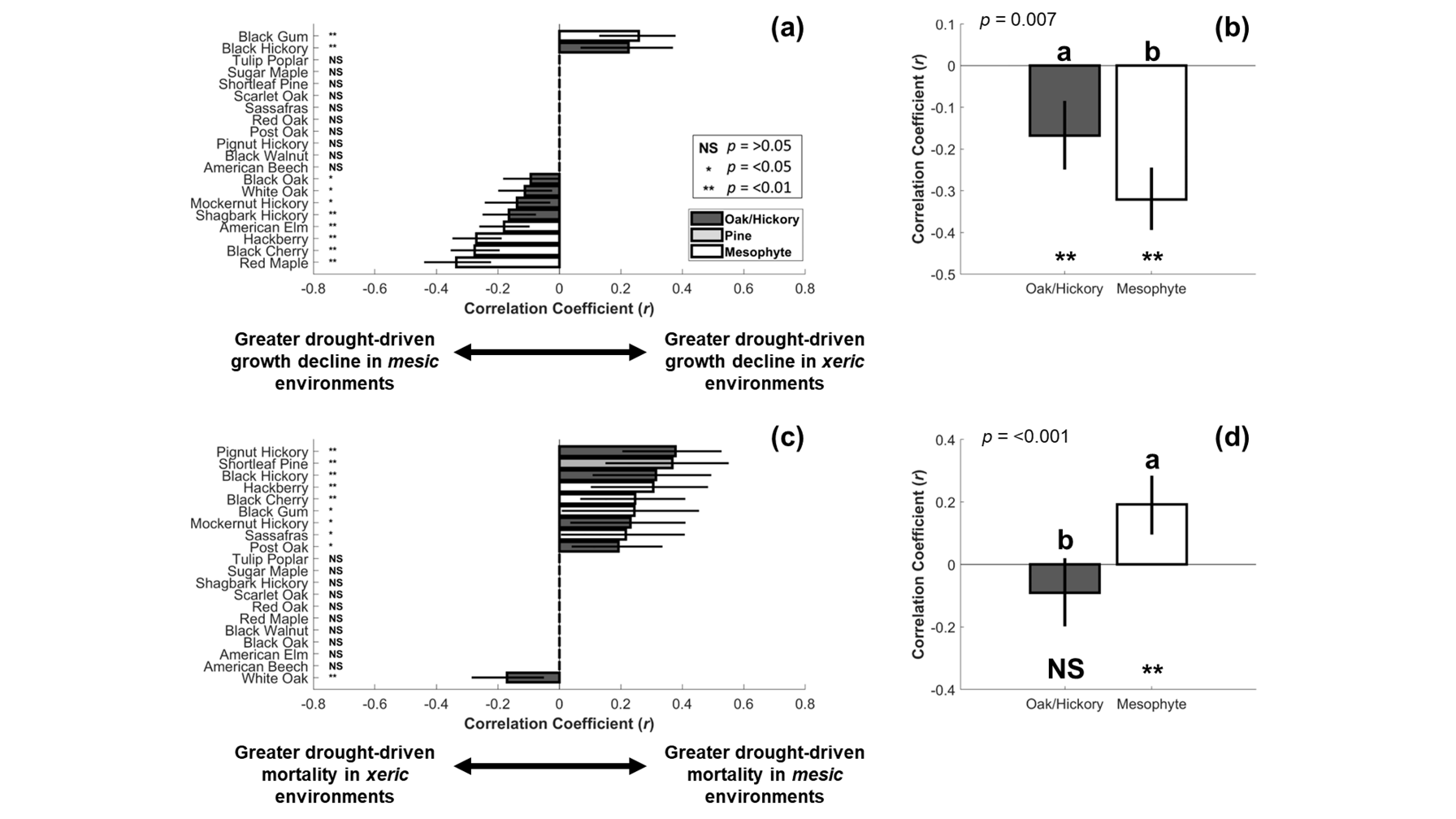


**Figure S6.** The iterative growth comparisons ($g_{r}$) between individual species pairs at the hexagon-level (Section 2.3 in the main text). Panels are pairwise difference comparisons between corrected relative growth rate of specific species (title) and those present in the same hexagons (x-axis). Green arrows denote the title species had greater growth following drought, a red arrow denotes the title species had reduced growth following drought, and ‘$\times$’ denotes both species experienced similar growth responses. Significant differences in responses were determined by one-sample *t*-test at the *α* = 0.05. Species codes are listed in Table 1 in the main text.


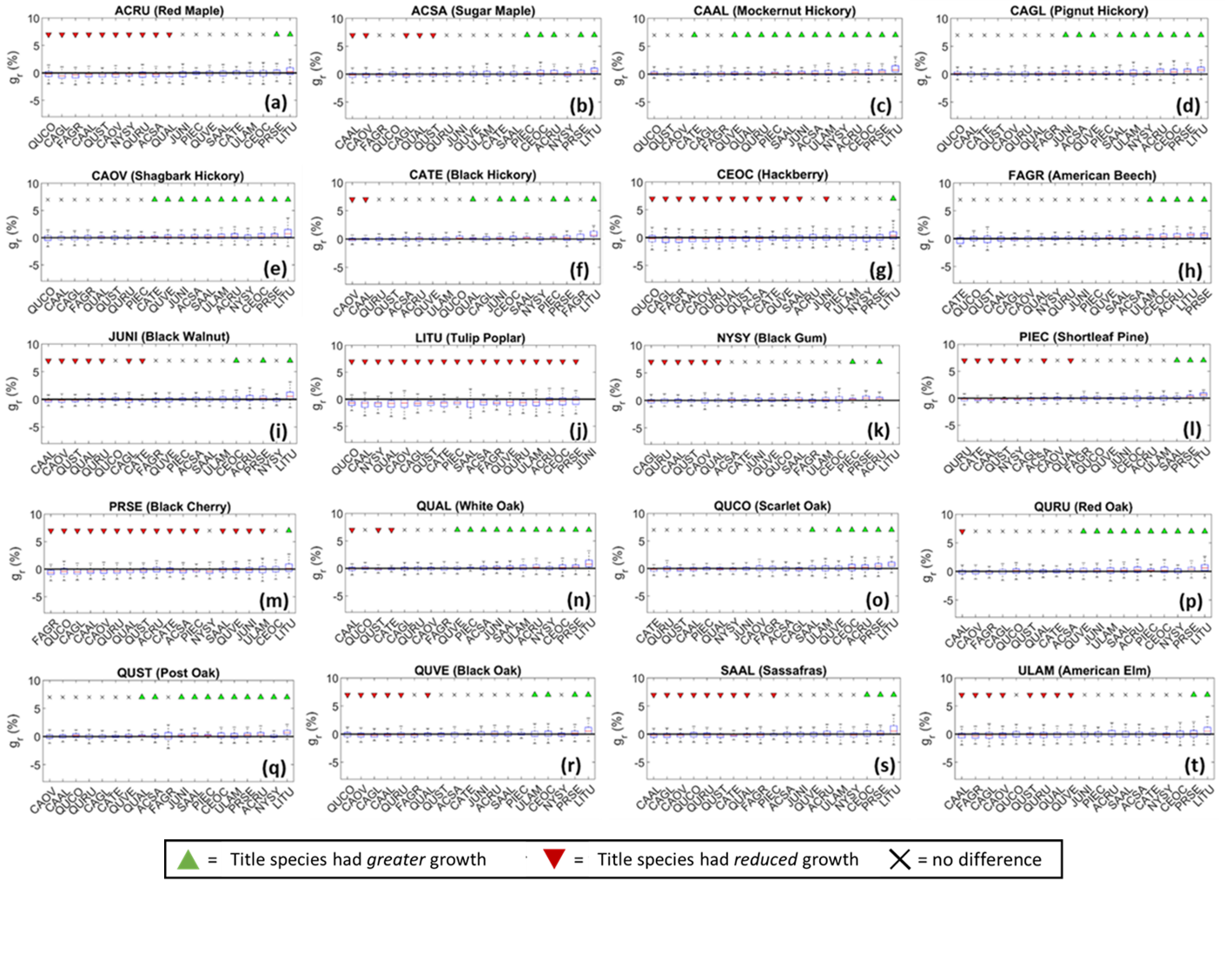


**Figure S7.** The iterative stem loss comparisons ($m_{r}$) between individual species pairs at the hexagon-level (Section 2.4 in the main text). Panels are pairwise difference comparisons between corrected stem loss rates of a specific species (title) and those present in the same hexagons (x-axis). Green arrows denote the title species had lower stem loss following drought, a red arrow denotes the title species had greater stem loss following drought, and ‘$\times$’ denotes both species experienced similar stem loss responses. Significant differences in responses were determined by one-sample *t*-test at the *α* = 0.05. Species codes are listed in Table 1 in the main text.


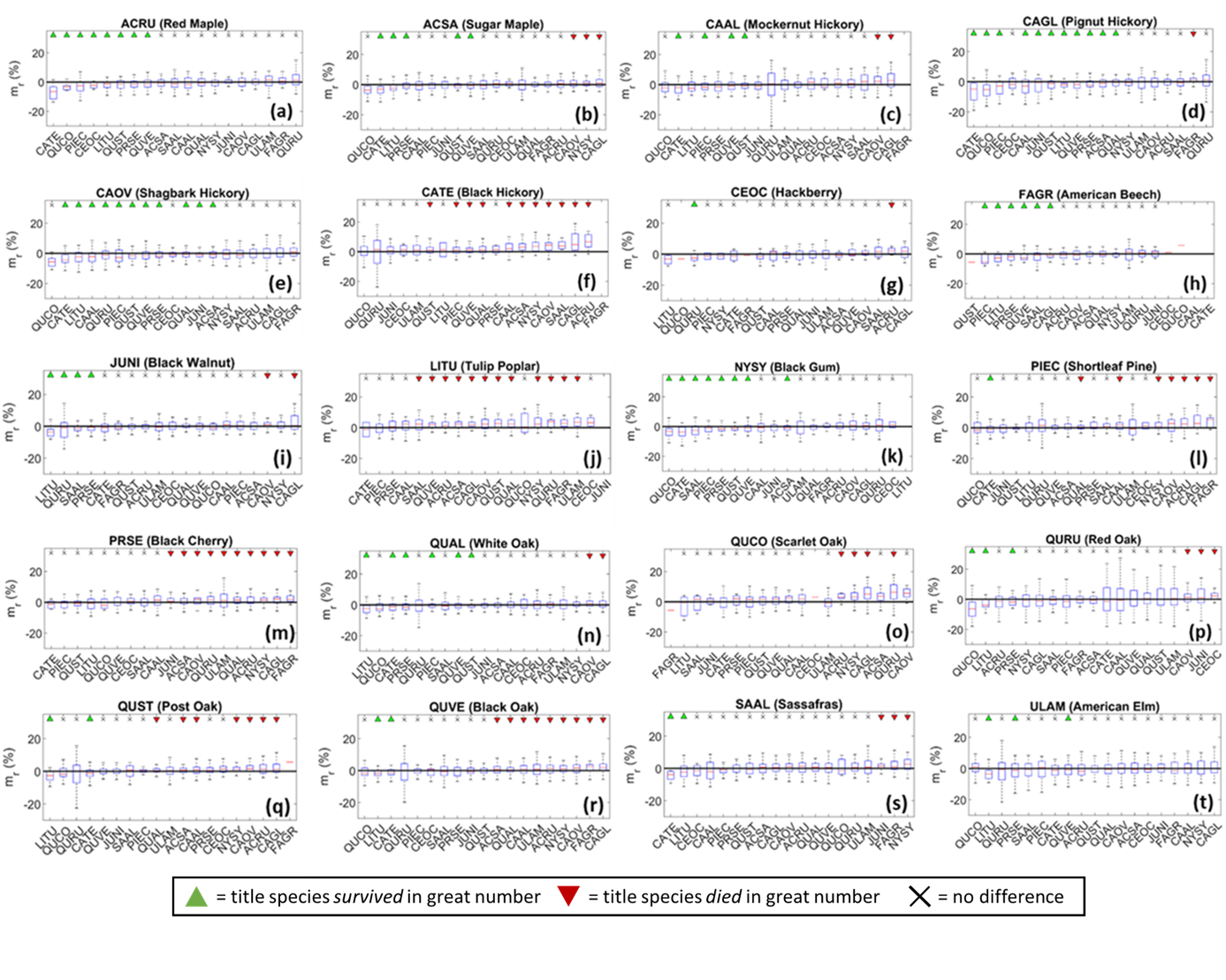


**Figure S8.** Relationship between relative growth sensitivity (%) and relative mortality sensitivity (%) among a lager tree subset, defined by species with a diameter at breast height greater than 20 cm. Panel (b) are *R*^2^ estimates from linear regression analyses evaluated across all species and functional groups (Table 1 in the main text). Lines in panel (a) are best fit from significant linear regressions (*α* = 0.05).


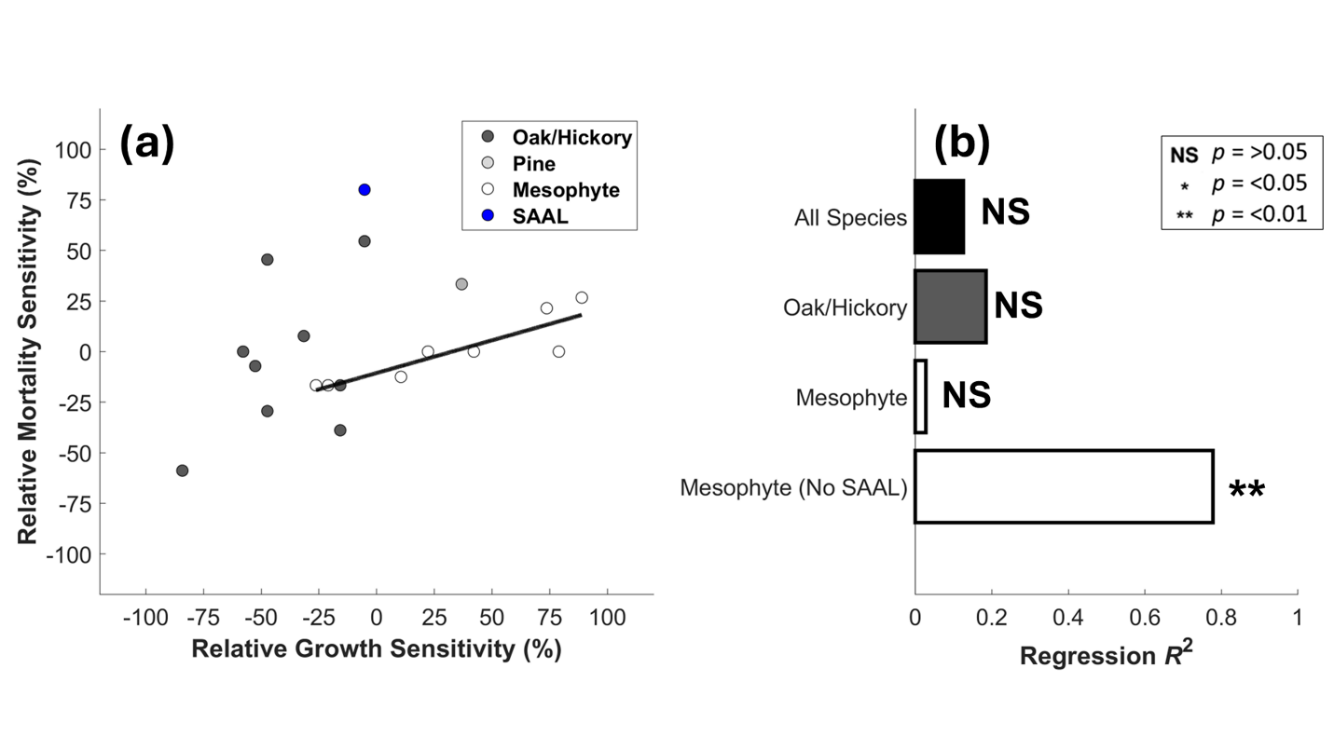


**Table S1.** FIA live stem counts (n) in each hexagon during the initial pre-drought period (from 2000-2005). Latitude and Longitude columns are the center coordinates of each hexagon (Hex ID). Species codes are listed in Table 1 in the main text.
